# Nucleosome Organization in Human Embryonic Stem Cells

**DOI:** 10.1371/journal.pone.0136314

**Published:** 2015-08-25

**Authors:** Puya G. Yazdi, Brian A. Pedersen, Jared F. Taylor, Omar S. Khattab, Yu-Han Chen, Yumay Chen, Steven E. Jacobsen, Ping H. Wang

**Affiliations:** 1 UC Irvine Diabetes Center, University of California Irvine, Irvine, California, United States of America; 2 Sue and Bill Gross Stem Cell Research Center, University of California Irvine, Irvine, California, United States of America; 3 Department of Medicine, University of California Irvine, Irvine, California, United States of America; 4 Department of Biological Chemistry, University of California Irvine, Irvine, California, United States of America; 5 Department of Physiology & Biophysics, University of California Irvine, Irvine, California, United States of America; 6 Department of Molecular, Cell and Developmental Biology, University of California Los Angeles, Los Angeles, California, United States of America; 7 Eli and Edythe Broad Center of Regenerative Medicine and Stem Cell Research, University of California Los Angeles, Los Angeles, California, United States of America; 8 Howard Hughes Medical Institute, University of California Los Angeles, Los Angeles, California, United States of America; Ludwig-Maximilians-Universität München, GERMANY

## Abstract

The fundamental repeating unit of eukaryotic chromatin is the nucleosome. Besides being involved in packaging DNA, nucleosome organization plays an important role in transcriptional regulation and cellular identity. Currently, there is much debate about the major determinants of the nucleosome architecture of a genome and its significance with little being known about its role in stem cells. To address these questions, we performed ultra-deep sequencing of nucleosomal DNA in two human embryonic stem cell lines and integrated our data with numerous epigenomic maps. Our analyses have revealed that the genome is a determinant of nucleosome organization with transcriptionally inactive regions characterized by a “ground state” of nucleosome profiles driven by underlying DNA sequences. DNA sequence preferences are associated with heterogeneous chromatin organization around transcription start sites. Transcription, histone modifications, and DNA methylation alter this “ground state” by having distinct effects on both nucleosome positioning and occupancy. As the transcriptional rate increases, nucleosomes become better positioned. Exons transcribed and included in the final spliced mRNA have distinct nucleosome profiles in comparison to exons not included at exon-exon junctions. Genes marked by the active modification H3K4m3 are characterized by lower nucleosome occupancy before the transcription start site compared to genes marked by the inactive modification H3K27m3, while bivalent domains, genes associated with both marks, lie exactly in the middle. Combinatorial patterns of epigenetic marks (chromatin states) are associated with unique nucleosome profiles. Nucleosome organization varies around transcription factor binding in enhancers versus promoters. DNA methylation is associated with increasing nucleosome occupancy and different types of methylations have distinct location preferences within the nucleosome core particle. Finally, computational analysis of nucleosome organization alone is sufficient to elucidate much of the circuitry of pluripotency. Our results, suggest that nucleosome organization is associated with numerous genomic and epigenomic processes and can be used to elucidate cellular identity.

## Introduction

Pluripotent stem cells hold great promise in regenerative medicine due to their ability to differentiate into all three germ layers: endoderm, mesoderm, and ectoderm. Human pluripotent stem cells can be divided into embryonic stem cells (hESC), which are derived from the inner cell mass of a blastocyst, and induced pluripotent stem cells (iPSC), which are generated or “reprogrammed” directly from somatic cells[1, 2]. To fully develop the possible therapeutic potential of stem cells, considerable research has been undertaken to study the role epigenetic modifications play in maintaining pluripotency and inducing differentiation. Additionally, recent work has demonstrated that while somatic and pluripotent cells share many similar epigenomic characteristics, there are unique features in the epigenome of embryonic stem cells[3–11]. While much of this work has focused on DNA methylation and chromatin modifications, epigenomic analysis of the primary unit of chromatin, the nucleosome, is scarce.

In eukaryotes, DNA is packaged into chromatin whose fundamental repeating unit is the nucleosome. The nucleosome is comprised of two copies of each of the core histones (H2A, H2B, H3, and H4) wrapped by 147 base pairs (bp) of DNA, with the symmetrical center being called the dyad[12]. Besides being involved in packaging DNA, nucleosome positioning (the genomic location of nucleosomes), nucleosome occupancy (how enriched a genomic location is for nucleosomes), and epigenetic modifications (post-translational modifications of histone proteins and DNA methylation) are thought to play a role in development, transcriptional regulation, cellular identity, evolution, and human disease[13–21]. Analyses in model organisms and humans have revealed that the nucleosome organization of a genome is affected by such diverse factors as underlying DNA sequences, nucleosome remodelers, protein binding, and the transcriptional machinery[13–18, 22–31]. Currently there is considerable debate about the roles and extent these factors play, especially in humans compared to yeast[32–36]. Furthermore, to the best of our knowledge, no one has generated genome-wide maps of nucleosomes in hESC and analyzed its potential role in pluripotency. To begin addressing these questions, we paired-end sequenced Micrococcal Nuclease (MNase) digested DNA from H1 and H9 human embryonic stem cells (hESC), yielding 180x and 70x depth of coverage of the human genome, respectively. A nucleosome occupancy score (NOS) map at single bp resolution without smoothing was calculated and used to call nucleosomes (Methods)[37]. The same processing was performed on ten other non-hESC datasets including one *in vitro* (IV) dataset, derived by reconstituting recombinant histones with genomic DNA from human granulocytes as a measure of the purely sequence driven component of nucleosome organization[17]. Additionally, nucleosome data was analyzed against a diverse set of epigenomic and genomic features[20, 38–40]. Finally, nucleosome architecture alone was used to predict transcription factor binding sites.

## Results

### Nucleosome map generation

H1 and H9 human embryonic stem cells were utilized to generate paired-end MNase-Seq data. After MNase digestion, nucleosomal DNA was visualized on a 2% agarose gel to assess for laddering of mono-, di- and tri-nucleosomal fragments (S1A Fig). Densitometry of the nuclesomal DNA from an image captured with a UV light box from one representative gel demonstrated that the band corresponding to mononucleosomal DNA (~150bp) was 71% of total DNA (S1A Fig). As can be seen by the DarkReader images and our methods, we titrated various amounts of MNase digestion, corresponding to 70–90% mononucleosomal DNA since this is considered the ideal amount of digestion[14–17]. As UV light can cause cross-linking of DNA and diminish the quality of DNA, the DNA used to make the next-generation sequencing libraries was visualized with a DarkReader (blue LED transilluminator) (S1B Fig). Two biological replicates for both H1 and H9 were performed, each consisting of six technical replicates for H1 and two technical replicates for H9. The two biological replicates were from two separate next-generation DNA sequencing runs (R51 and R54). 2,586,825,651 and 869,318,927 raw paired-end reads from H1 and H9 cells were sequenced, respectively. This corresponds to an average depth of coverage of approximately 180x for the H1 cell line and approximately 70x for the H9 cell line. The total number of raw reads and the alignment data is shown in S1 Table.

We then compared all technical and biological replicates. Specifically, we performed genome-wide Pearson correlation coefficients (PCC) of the BAM files from each replicate. Please see S2 and S3 Tables, as well as S2 and S3 Figs for the data presented as a table and as a heatmap, respectively. This analysis compares all individual H1 replicates to one another, between sequencing runs, to the pooled H1 datasets, and includes a comparison of the sequencing runs. The same was also done for the H9 replicates. Additionally, the pooled H1 and H9 datasets from both sequencing runs were compared. The minimum PCC between all individual H1 and H9 replicates is 0.892 and 0.982, respectively. The average PCC for this comparison for all H1 replicates was 0.969 and for all H9 replicates was 0.989. Finally, the pooled H1 and pooled H9 datasets were directly compared and had a PCC of 0.961. Based on the high similarity between the H1 replicates, all H1 replicates were combined for downstream analysis according to ENCODE guidelines[38]. As this was also true for H9, they were also combined. The DANPOS algorithm has previously been shown to outperform other nucleosome positioning software and was thus utilized to process all sequencing datasets[37]. After processing the datasets through the DANPOS algorithm, a distribution of nucleosomal fragment sizes for each dataset were compared (S4 Fig). The average fragment size for all datasets was 153.7 bp. The fragment sizes for the paired-end datasets (H1, H9, GM18507, GM18508, GM18516, GM18522, GM19193, GM19238, GM19239) are indeed consistent with mononucleosomal DNA and demonstrate a typical size distribution.

### Sequence driven nucleosome organization

**Fig 1:**
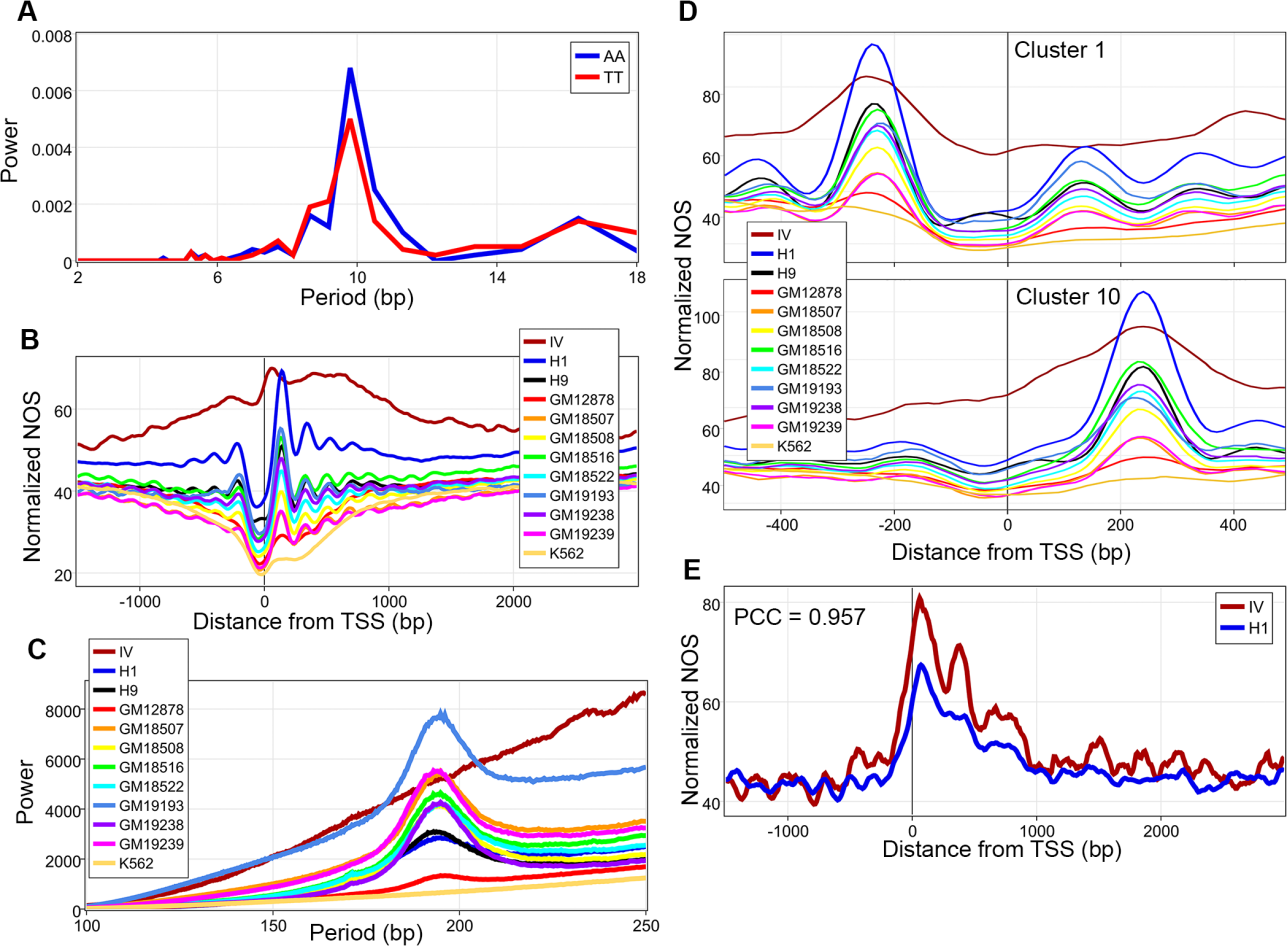
Sequence driven nucleosome organization. **A**, Fast Fourier transforms (FFT) of AA and TT dinucleotide frequencies through the nucleosome core particle were calculated revealing ~10 bp periodicities. **B**, Normalized nucleosome occupancy scores (NOS) from 12 datasets averaged over 13,912 unique transcription start sites (TSS). **C**, Genome-wide FFT of all 12 datasets showing lack of arrayed nucleosomes in the *in vitro* (IV) dataset. **D**, Normalized NOS for all 12 datasets based on *k*-means clustering of the H1 data, demonstrating that the location of the major peak around TSS is determined by underlying DNA sequences, see S9 Fig for remaining clusters. **E**, IV and H1 NOS were calculated against inactive TSS, the two datasets were highly correlated with a Pearson’s correlation coefficient (PCC) of 0.957.

We then turned our attention to the role of DNA sequences in nucleosome organization. While considerable work has been done on these features in model organisms, we sought to ascertain if these features are observed in H1 and H9 hESCs. Nucleosomes are enriched for G/Cs and depleted for A/Ts (S5A and S6A Figs). Additionally, AA/TT dinucleotides show ~10 bp periodicities (by fast Fourier transforms (FFT)) confirming that sequence preferences are conserved across eukaryotes (Fig 1A and S6B–S6D Fig)[13, 22, 23]. Interestingly, all other dinucleotides also demonstrated small ~10 bp periodicities (S5B–S5D and S6B–S6D Figs). NOS maps for all 12 datasets were analyzed against 13,912 high confidence protein coding gene coordinates with unique transcription start sites (TSS) (Methods). IV nucleosomes failed to produce well-positioned arrays, a finding confirmed by genome-wide FFT, in line with recent work that shows this is a role of nucleosome remodelers (Fig 1B and 1C and S7 Fig)[18, 26, 27]. Overall, we can conclude that sequence driven components of nucleosome architecture are independent of cell type.

Recently, clustering analysis has shown that chromatin architecture around TSS is heterogeneous[28]. *K*-means clustering of the H1 NOS around TSS revealed 10 clusters that have either a well-positioned downstream or upstream nucleosome, corroborating previous analysis (S8 Fig). Intrigued by what was driving their location, we hypothesized that it could be underlying DNA sequences. To test this, all datasets were analyzed against the coordinates for the 10 different clusters derived from H1. In all 10 clusters, the location of the predominant peak for nucleosome occupancy in each cell line was similar(Fig 1D and S9 Fig). To better visualize this finding, we plotted the location of maximum NOS for all cell lines for all 10 clusters(S10 Fig). The location of the points in this plot are representative of the location of the dyads for the nucleosomes with the highest occupancy within the entire cluster for a given cell line. Based on the clustering analysis, we hypothesized that DNA sequences were a primary driver of nucleosome positions in the absence of transcription. Specifically, the contribution of the underlying DNA sequence should be greatest in transcriptionally silent regions. Furthermore, nucleosomes are strand independent while transcription is strand specific, and a large portion of the genome is transcribed, regardless of its functional significance[38]. Hence, to accurately determine transcriptionally silent TSS we created a total RNA signal map (RNA-Signal) at single bp resolution by adding the signal from both strands[38]. Based on this map, silent genes were defined and analyzed for both the H1 and IV NOS revealing that the two were highly correlated (PCC = 0.957, Fig 1E). By accounting for known H1 structural variants, the IV and H1 datasets show a genome-wide PCC of 0.695. This suggests that underlying DNA sequences around TSS are highly correlated with nucleosome organization and could create a “ground state” of nucleosome architecture in such regions. Additionally, our genome-wide mononucleotide frequencies and FFT analyses of dinucleotide frequencies within the nucleosome core particle, in conjunction with the nucleosome occupancy correlations between the IV and H1 datasets, implicate underlying DNA sequences play a role in determining nucleosome organization.

### Epigenetic regulation of nucleosomes

**Fig 2:**
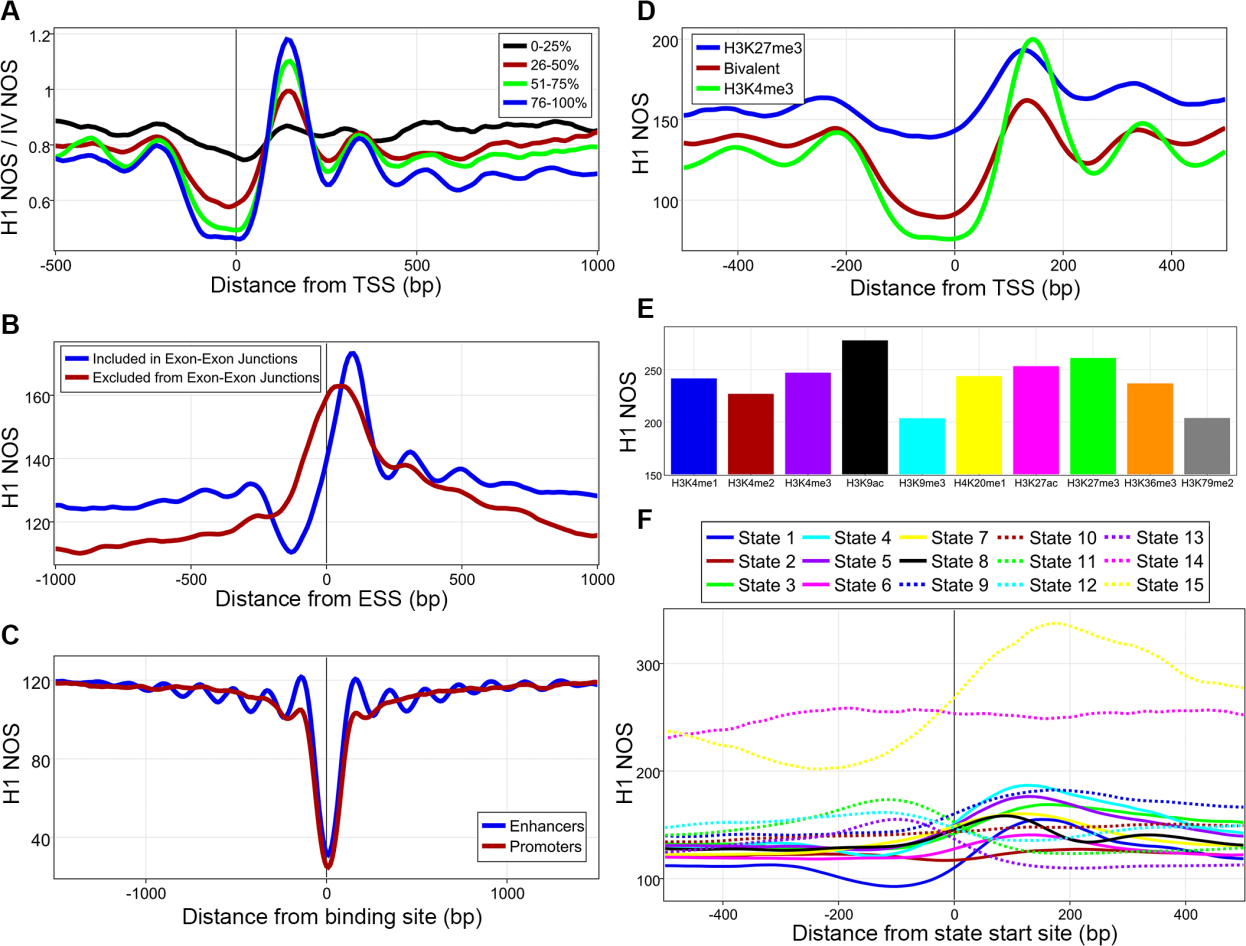
Epigenetic regulation of nucleosomes. **A**, 13,912 genes were divided into quartiles based on total RNA-Expression from both strands with 0% being lowest total expression and 100% highest expression. **B**, Nucleosome occupancy score (NOS) plotted against exon start sites (ESS) from exons included in exon-exon junctions and those excluded. **C**, H1 NOS averaged around transcription factor binding sites at active promoters and active enhancers. **D**, NOS plotted for genes marked by H3K4me3, H3K27me3, or both (bivalent) in their promoters. **E**, Average NOS for whole nucleosomes found with one of 10 histone-modifications, all comparisons were statistically significant. **F**, H1 NOS plotted against 15 different chromatin state start sites, see S11 Fig for definitions.

Next, we sought to address how hESC specific transcription, transcription factor binding, and histone post-translational modifications can alter this “ground state”[38]. We used our RNA-Signal to break up our gene list into quartiles based on total RNA expression and analyzed H1 NOS map against these coordinates and divided the signal by the IV NOS for that gene to accurately quantify how transcription changes the “ground state”[38]. As the transcriptional rate increases the nucleosome depleted region (NDR) becomes less occupied, the +1 nucleosome becomes better positioned with an increased peak, and the nucleosomes become better arrayed, demonstrating how the transcriptional machinery, most likely along with remodelers, work to space nucleosomes and alter the compaction of the chromatin (Fig 2A)[18]. Since the IV dataset might be biased due to being generated with very low assembly degrees (one nucleosome per 850 bp) and on rather short DNA fragments, we repeated this analysis without normalization against the IV dataset. Though less easily discernible, these same findings were found in this analysis (data not shown). We then computed NOS around exons included and excluded in exon-exon splice junctions and found that nucleosomes cover much more of the junction in excluded exons, hinting at their possible role in alternative splicing (Fig 2B). We also find that nucleosome architecture at transcription factor binding sites is only arrayed at active enhancers and not at active promoters (Fig 2C). Coordinates for enhancers and promoter were from the ENCODE dataset[38]. Active sites were defined as those for which the DNase-Seq signal was high, a ChiP-Seq peak was called for a transcription factor, and NOS was low in H1 cells. It must be stated that transcription factor binding sites at active promoters are not necessarily in close proximity to its associated TSS. In fact, the median distance between the transcription factor binding site at active promoters and its associated TSS is 1686 bp for the H1 dataset. We then looked at how histone post-translational modifications can affect nucleosomes. Genes marked by the inactive modification H3K27me3 in their promoters have a higher overall NOS and are less depleted at the NDR compared to genes marked by the active modification H3K4me3 while bivalent genes lie in the middle of the two antagonistic marks (Fig 2D)[41]. Additionally, the location of the +1 nucleosome shifted further downstream, 7 bp from inactive to bivalent and 10 bp from bivalent to active. The average NOS for nucleosomes marked by one of ten histone marks was calculated and showed statistically significant differences for all comparisons (Kruskall-Wallis followed by Mann-Whitney-Wilcoxon with Bonferroni correction, Fig 2E and S5 Table). Finally, NOS was plotted against 15 predicted chromatin state start sites (S11 Fig for definitions) and show that combinatorial patterns of epigenetic marks are correlated with unique and specific nucleosome architectures (Fig 2F and S11 Fig)[20]. Chromatin states are defined computationally by a collection of epigenetic marks. As such, certain states are probably more readily defined than others. Additionally, as these locations are defined by a start site of a region of DNA and not by a transcription factor which binds to the middle of this region, it is impossible to directly compare these chromatin state figures with traditional figures of nucleosomes around promoters, enhancers, and TSS which are generated around the middle of a region. That being said, we did find it interesting that the insulators are associated with well arrayed nucleosomes regardless of these limitations. Overall, we can surmise that active marks and activation of the transcriptional machinery is associated with lower nucleosome occupancy and better-arrayed nucleosomes.

### DNA methylation and nucleosomes

**Fig 3:**
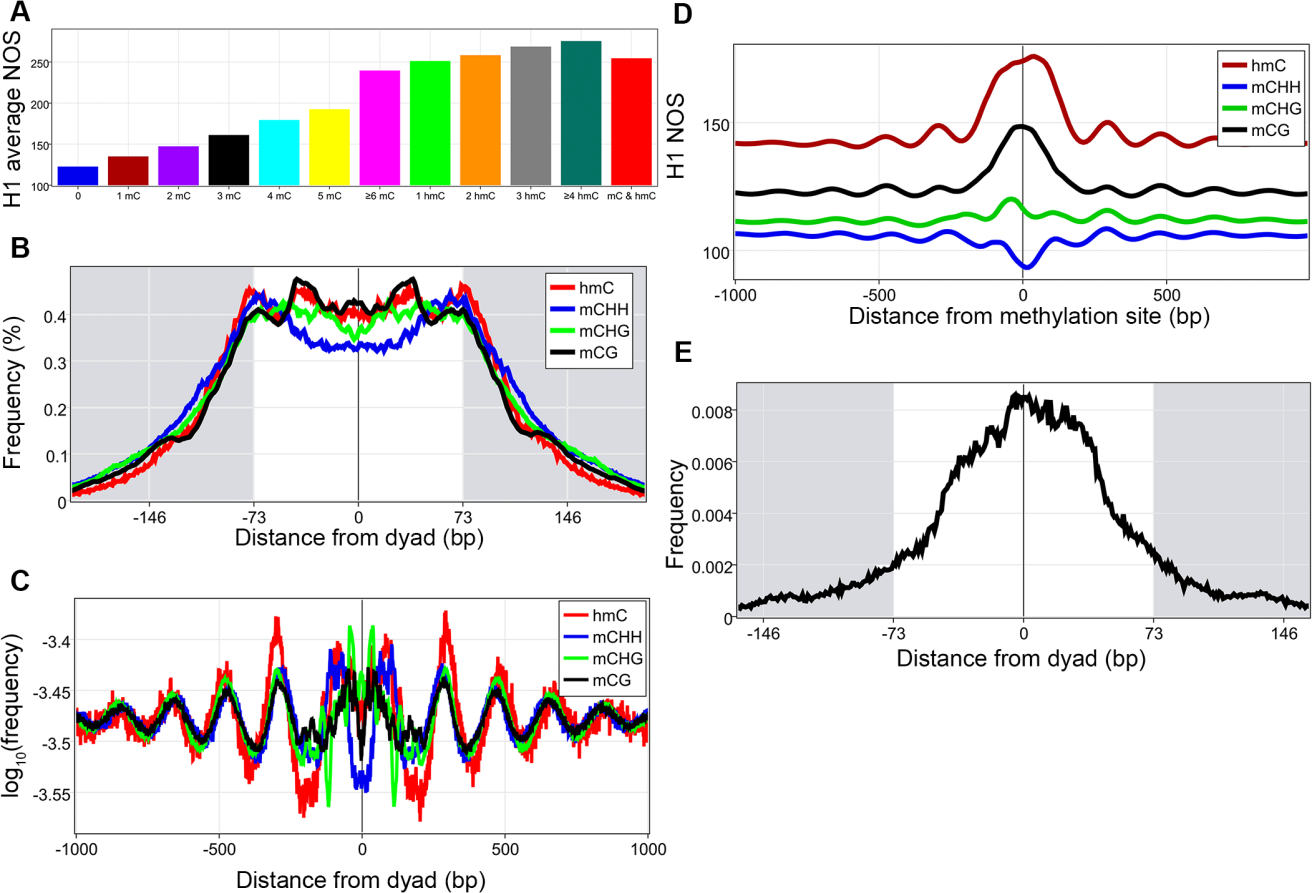
DNA methylation and nucleosomes. **A**, Nucleosomes were divided based on the type and number of methylations found in them and the average nucleosome occupancy score (NOS) was calculated for each group, all comparisons were statistically significant. **B**, The distance from the dyad to the closest methylation was calculated for all mCG, mCHH, mCHG, and hmC separately and converted to frequency percentages. **C**, Plot of all DNA methylations around nucleosome dyads. **D**, NOS were calculated around all mCG, mCHG, mCHH, and hmC sites. **E**, Plot of mCG frequency to closest dyad for mCGs associated with NOS above 190, demonstrating their enrichment toward the dyad.

We then addressed how DNA methylation affects nucleosome occupancy[39, 40, 42]. Increasing methylated cytosines found in a nucleosome is associated with an increased average nucleosome occupancy (statistically significant by a Kruskall-Wallis followed by Mann-Whitney-Wilcoxon with Bonferroni correction, Fig 3A and S5 Table). Studies in plants have shown that methylated cytosines are enriched within nucleosomes and display ~10 bp periodicity[43]. To investigate these findings in humans, the distance from each of the four types of methylations (three types of 5-methylcytosine (mCG, mCHG, mCHH, where H = A,T,or C), and 5-hydroxymethylcytosine (hmC)) to the nearest dyad was plotted, revealing that the four types of methylations have distinct location preferences within the nucleosome (Fig 3B). Interestingly, mCG is enriched at around +/- 40 bp and around the dyad, the three locations that have the strongest DNA nucleosome binding, providing a possible mechanism whereby DNA methylations can increase nucleosome occupancy. FFTs of the four different methylations within the nucleosome core revealed the periodicity of the signal (S12 Fig)[44]. All methylations against dyads were plotted revealing that on a genome-wide level, methylations are found in a periodic pattern around nucleosome dyads (Fig 3C). We hypothesized that this periodic methylation pattern might then be associated with periodic nucleosomes and by plotting H1 NOS around methylation sites we demonstrate this with the caveat that mCHHs are associated with a decreased NOS (Fig 3D). Finally, we became intrigued by the possibility that mCGs associated with higher NOS (those above the average peak signal of 190) might have a greater enrichment closer to the dyad thereby increasing the DNA-histone interaction, which our results demonstrate (Fig 3E). This brings about the intriguing possibility that DNA methylation can deactivate genes by creating tightly bound nucleosomes that are an impediment to transcriptional machinery.

### Nucleosome architecture and the circuitry of pluripotency

**Fig 4:**
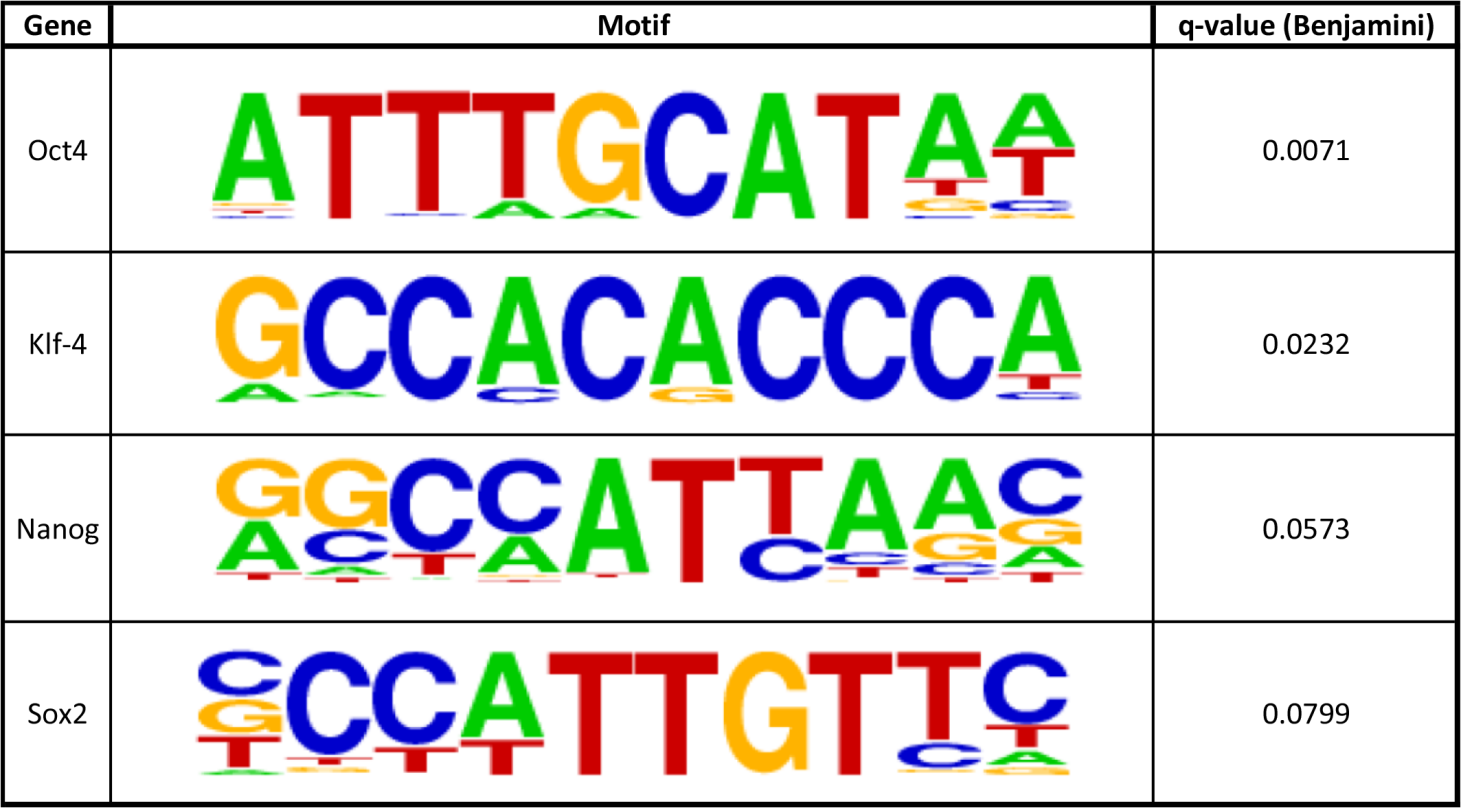
Computationally derived active enhancer sites. HOMER motifs with their associated gene at enhancer binding site locations, as defined by surrounding nucleosome positioning (Methods). Included in the table are genes that maintain the pluripotent state.

We then turned our attention to the possibility that genome-wide nucleosome maps could be used to deduce the circuitry of transcription factors driving the cell state. Our data along with previous work has demonstrated that transcription factors turn on genes by binding to enhancers and promoters and displacing nucleosomes in the process. This process creates well-defined nucleosome architectures: a missing nucleosome surrounded by two bound nucleosomes that are relatively well-positioned. Hence, we hypothesized that by scanning our nucleosome map for these patterns and then integrating the resulting DNA sequences with motif discovery tools we might be able to ascertain some of the transcription factors that drive the circuitry of pluripotency. Our computational approach was able to predict that Oct4, Sox2, KLF4, and Nanog, classic transcription factors used for reprogramming and believed to be driving the circuitry of pluripotency, are active in our cell line based on nucleosome analysis alone (Fig 4, S13 Fig)[1, 45–47]. Based on this data, it seems plausible that nucleosome analysis could be used as a first step in reprogramming or transdifferentiating different cell types by helping generate a list of active transcription factors driving that cell’s circuitry.

## Discussion

By integrating our H1, H9, and published somatic cell *in vivo* data with *in vitro* data, we set out to determine how and in what ways underlying DNA sequences are associated with nucleosome organization and if these patterns were similar in pluripotent and somatic cells. The IV dataset was created by reconstituting recombinant histones with DNA from human granulocytes in ~1:1 molar ratio and hence nucleosome occupancy variation across the genome is being driven by underlying DNA sequence preferences alone[17]. Additionally, since ~10 bp periodicity cannot be easily discerned, we followed previously establish methods and utilized a FFT to determine the periodicity, if any[17, 43]. A Fourier transform is a mathematical method, with many different applications, that converts a signal in space into a combination of pure frequencies. As such, FFTs were performed for each dinucleotide to more precisely determine if a periodicity (1/frequency) existed, and if so what it is for each dinucleotide within the nucleosome core particle (Fig 1A, S5D and S6D Figs). Our data corroborates studies in yeast and a recent work in humans that has shown that nucleosomal DNA demonstrates an AA/TT ~10 bp periodicities and is enriched for G/C content (Fig 1A, S5A, S6A and S6D Figs)[13, 22, 23]. Furthermore, we can conclude that on a global level, nucleosome architecture is similar in both somatic and pluripotent cells. This is evidenced by the similarity of the nucleosome architecture around the TSS in all of the datasets (Fig 1D and 1E, S9 and S10 Figs). This is further corroborated by the genome-wide PCC of 0.695 between NOS maps for H1 and IV datasets. These findings suggest that nucleosome organization could be driven by underlying DNA sequences in transcriptionally silent regions(though we cannot rule out that the underlying molecular biology could have biased the correlations), which can lead us to hypothesize that the genome drives a primary organization of nucleosome architecture, which the epigenome and transcription can alter in a cell-specific manner.

Taking advantage of the richness of the available ENCODE data, we integrated our H1 data with numerous epigenomic maps[38]. First, we demonstrated how spacing of nucleosomes and ordered arrays around TSS is highly correlated with the transcriptional rate (Fig 2A). Since previous work has shown that nucleosome remodelers are involved in spacing nucleosomes, it appears that remodelers and the transcriptional machinery work together to create the classic nucleosome organization[18, 26, 27]. It is also tempting to speculate that perhaps histone post-translational modifications can be read by nucleosome remodelers and this would allow for a fine-tuning of directionality and needed spacing for transcription. The variable nucleosome architecture around exons falls in line with recent work that has shown that chromatin plays a role in alternative splicing (Fig 2B)[48–50]. Interestingly, transcription factor binding at enhancers and promoters created different chromatin architectures (Fig 2C). Since the classic arrayed pattern was only observed at enhancers, it is tantalizing to hypothesize that perhaps nucleosome organization around active enhancers is involved in three-dimensional structural changes and possibly DNA looping. Both nucleosome positioning and occupancy differences were associated with different histone post-translational modifications and combinatorial patterns of these marks were also associated with specific nucleosome organizations (Fig 2D–2F and S5 Table)[20, 41]. These findings, taken together with work that has shown variation in nucleosome repeat length is associated with different histone post-translational modifications, leads us to speculate that an important function of histone post-translational modifications could be to alter nucleosome organization[17, 25].

DNA methylation represents the final epigenetic modification that we analyzed. Our findings confirmed work done in plants that has shown enrichment for methylated cytosines within nucleosomes and display periodicities within the nucleosome core particle (Fig 3A and 3B and S5 Table and S12 Fig)[43]. Most interestingly, is that all four types of methylations are associated with distinct location preferences within the core particle. It will be interesting going forward to determine if these location preference differences have important functional significance, for example being used to fine tune nucleosome location during differentiation. Additionally, the CG methylation preferences around ± 40 bp most directly tie in methylation to increasing nucleosome occupancy since the three locations that have the highest potential nucleosome DNA binding capacity, dyad and ± 40bp, are the three highest enriched for CG methylations[44]. This is further corroborated by our findings of increasing nucleosome occupancy with an increasing number of methylations found within the core particle. These findings suggest that a role of DNA methylation is to alter nucleosome organization by increasing nucleosome occupancy, which can lead to deactivation. However, it must be stated that patterns of DNA methylations may not cause changes in nucleosome organization but could instead result from it.

We finally turned our attention toward determining if genome-wide nucleosome maps in combination with computational motif discovery tools alone were enough to determine which transcription factors are active in a cell type. It has been known for some time that DNase I hypersensitivity sites, which correspond to nucleosome free regions, can be used to find possible transcription factor binding sites. We sought to ask if nucleosome organization was sufficient to find these sites. By utilizing nucleosome architecture alone, our computational approach correctly predicted that the master regulators of pluripotency (OCT4, Nanog, Sox2, and KLF4) are active in H1 hESCs (Fig 4). Additionally, our approach also found other potential transcription factors that could be active in H1 stem cells, such as Tcf12, Mef2c, and HNF6 (S13 Fig). It will be interesting to see how much of a role, if any, these other factors play in maintaining pluripotency. Furthermore, this approach represents a proof of concept and further work can be done to fine-tune this approach. Since it has been shown that both DNase I and MNase maps introduce their own biases, it would be an interesting follow up application to integrate these two maps with motif analysis to discern if this approach could lead to a more robust platform for transcription factor discovery.

## Methods

### Cell culture

The UC Irvine Human Stem Cell Research Oversight Committee (UCI hSCRO) approved the use of human embryonic stem cells in this study. H1 and H9 human embryonic stem cell lines were purchased from WiCell Research Institute, Inc. These are some of the first ever human embryonic stem cell lines ever derived and are approved by the NIH Human Embryonic Stem Cell Registry (http://grants.nih.gov/stem_cells/registry/current.htm)[2]. The NIH Registration Numbers for H1 and H9 human embryonic stem cells are 0043 and 0062, respectively. Feeder free cultures of H1 and H9 human embryonic stem cells were grown and passaged in mTeSR 1 (STEMCELL Technologies Inc) as previously described and in accordance with ENCODE protocols to ease comparison to published ENCODE datasets[38]. In total, approximately 100 million H1 and H9 cells corresponding to passages 33–35 were used in subsequent experiments.

### Generation of mono-nucleosomal DNA sequenced reads

H1 and H9 cells were subjected to MNase digestion by use of the EZ Nucleosomal DNA Kit (Zymo Research) in accordance with the manufacturer’s protocol. The ideal digestion should yield approximately 80% mono-nucleosomal DNA[14–17]. In order to extract both easily digested nucleosomes and less digestible ones, we titrated the time of digestion in multiple replicates to yield 70% to 90% mono-nucleosomal DNA, with the average being 80% from all replicates combined. We then prepared paired-end libraries from this total mono-nucleosomal DNA with use of the Illumina Paired-End DNA Sample Prep Kit according to the manufacturer’s instructions with one exception. In order to reduce potential PCR amplification bias, we performed two separate PCR reaction steps and combined the product of the two reactions[42, 51]. The libraries were then sequenced using PE54 chemistry on the Illumina HiSeq2000 in replicate on two flow cells (R51 and R54). Two biological replicates for both H1 and H9 were performed, each consisting of six technical replicates for H1 and two technical replicates for H9.

### Alignment and processing of nucleosome maps

#### Alignment

Paired-end nucleosomal sequencing data from H1 and H9 cells from R54 was aligned to the hg19 reference genome using the Bowtie2 algorithm on default settings[52]. Data from R51 was processed similarly with the exception that 25 bases from the 3' end were removed as these final cycles produced low Q-scores which caused excess reads to not align. Additionally, we processed raw sequencing paired-end nucleosome data on the same default settings using Bowtie2 for GM18507, GM18508, GM18516, GM18522, GM19193, GM19238, GM19239[23]. Raw sequencing data for the IV dataset was downloaded and aligned in colorspace by use of default settings on Bowtie[17, 53].

#### Processing

All aligned data was processed using SAMtools to yield merged BAM files[54]. Finally, processed BAM files for K562 and GM12878 were downloaded from the ENCODE portal on the UCSC genome browser and merged[55].

#### Validation

BAM files from each H1 and H9 lane of sequencing data, as well as the merged datasets were compared using the deepTools bamCorrelate function[56]. The settings for bamCorrelate were as follows:—binSize 100,—corMethod Pearson,—outFileCorMatrix [H1 Table | H9 Table | H1-H9 Table],—plotFileFormat [H1 Heatmap | H9 Heatmap | H1-H9 Heatmap](S2 and S3 Tables, S2 and S3 Figs).

### Nucleosome occupancy score map generation and calling nucleosomes

BAM files were run through the DANPOS algorithm in which reads were clonally cut to remove potential PCR amplification bias, smoothed, and adjusted for nucleosome size to enhance signal to noise ratio, resulting in a nucleosome occupancy score (NOS) for each base in the human genome[37]. DANPOS settings were as follows for all paired-end datasets (H1, H9, GM18507, GM18508, GM18516, GM18522, GM19193, GM19238, GM19239):-d 150,-a 1,-k 1,-e 1,—paired 1. For single-read datasets (*in vitro*, K562, GM12878), the following DANPOS settings were used:-d 150,-a 1,-e 1,-k 1.-d 150 denoted setting the minimal distance between nucleosome dyads to 150 bp. The distance between dyads was set to 150 bp as the average fragment size from our H1 paired-end sequencing dataset was 151 bp (corresponding to 75 bp on either side of a dyad).-a 1 set the resolution of the NOS maps at a single bp and thus obviated any further downstream signal smoothing. The setting-e 1 allows for an edge-finding step to be taken, which estimates the edges of the predicted nucleosomes.-k 1 led to all data from intermediate steps being saved.—paired 1 indicated that the input BAM files were from paired-end sequencing data. This single base pair resolution NOS map was used to call best-fit nucleosomes with a corresponding average NOS so long as there was a minimum distance of 150 bp between two nucleosomes. Additionally, for all comparisons between different nucleosomal datasets, the NOS were normalized. We also generated NOS and called nucleosomes for the H1 dataset corrected for MNase digestion bias with use of a genomic control and found no significant differences in sequence preference analyses (data not shown)[22, 23, 43, 57]. For all subsequent analyses we used our original NOS map and called nucleosomes.

### General software used for analysis

Operations on genomic intervals were performed using BEDTools[58]. Fast Fourier transforms were done using MATLAB. Statistics were done in R. Heatmaps were generated with the Gitools software package[59]. Additionally, we made use of in-house Python 2.7, C++, and shell scripts that are available upon request.

### Genomic annotations, DNA sequence preferences, clustering and total RNA-Signal generation

Mononucleotide and dinucleotide frequencies were computed by use of custom made Python and C++ programs. Gene coordinates were based on RefSeq coordinates that had at least 50% overlap with the consensus coding sequence (CCDS) gene coordinates[60, 61]. Additionally, we restricted the analysis to genes with unique transcription start sites, removing any duplicates. *K*-means clustering was performed as previously described[62, 63]. We initially chose a wide range of *k* values (data not shown) and used 10 as it yielded the clearest differences between clusters. Processed RNA sequence data was downloaded from ENCODE as bigWig files for the plus and minus strands[38]. The two signal files were normalized and added up to generate a single RNA-Signal file that we subsequently used to calculate transcriptionally silent genes and quantify total RNA-Expression of each gene from our initial list. Genome-wide Pearson’s correlation coefficients (PCC) were performed by binning the NOS sets every 10 bp after removing coordinates corresponding to structural variants in H1 as defined by ENCODE[38].

### Transcription factor binding, histone modifications, and chromatin states

Exon-exon junctions were downloaded from ENCODE[38]. We used ENCODE Affymetrix exon microarray data as an independent test to verify exon inclusion and exclusion in H1 transcripts. All enhancer and promoter coordinates were downloaded from ENCODE as were transcription factor binding sites and DNase-Seq signal. We called active enhancers and promoters as those that fell within one transcription factor binding site by ChIP-Seq data, had a high DNase-Seq signal and a low NOS. Encode histone-modification called peaks were used along with our called nucleosomes to assign called nucleosomes to one of ten corresponding modifications. Chromatin state start sites were downloaded from the UCSC genome-browser table.

### DNA methylation analysis

Called and processed methylation data was downloaded and converted to hg19 using the liftOver utility[39, 40, 55]. Additionally, all sites called as methylated cytosines that were subsequently shown to be hydroxymethylcytosines were removed from the methylcytosine coordinates.

### DNA motif analysis

Motif analysis was performed using HOMER with the following settings: Size 200, S 50, Len 6–14, Mis 3[45]. The input motif locations were determined by scanning the genome for the visual enhancer binding site motif (Fig 2C). Briefly, this is done by taking called nucleosomes from the DANPOS algorithm, and utilizing the NOS scores to locate the most well positioned nucleosomes with an intervening depleted region, that are also flanked by multiple additional well positioned nucleosomes, that also contain intervening depleted regions. The position of the intervening depleted region of the most well positioned nucleosomes was then analyzed user HOMER.

## Supporting Information

S1 Fig Nucleosomal DNA post-MNase treatment. **A**, MNase treated DNA resolved in a 2% agarose gel stained with ethidium bromide and visualized with a UV light source. **B**, MNase treated DNA resolved in a 2% agarose gel stained with ethidium bromide and visualized with a visible blue light (DarkReader Transilluminator).
(TIF)

S2 Fig Heatmap of H1 BAM comparison. Heatmap visualization with hierarchical clustering of the Pearson correlation coefficients performed for the H1 cell line as per S2 Table.
(TIF)

S3 Fig Heatmap of H9 BAM comparison. Heatmap visualization with hierarchical clustering of the Pearson correlation coefficients performed for the H9 cell line as per S3 Table.
(TIF)

S4 Fig Distribution of nucleosome fragment sizes. Fragment sizes were inferred through the use of the DANPOS algorithm.
(TIF)

S5 Fig Mono- and Dinucleotide frequency analysis for H1. **A**, Mononucleotide frequencies in relation to the dyad. **B**, Dinucleotide frequencies for AA, TT, CC, and GG in relation to the dyad. **C**, Dinucleotide frequencies for all 16 dinucleotides plotted in four panels (top to bottom). **D**, Fast Fourier transforms (FFT) for all dinucleotides (minus AA and TT, see Fig 1A) plotted in four panels (top to bottom).
(TIF)

S6 Fig Mono- and Dinucleotide frequency analysis for H9. **A**, Mononucleotide frequencies in relation to the dyad. **B**, Dinucleotide frequencies for AA, TT, CC, and GG in relation to the dyad. **C**, Dinucleotide frequencies for all 16 dinucleotides plotted in four panels (top to bottom). **D**, Fast Fourier transforms (FFT) for all dinucleotides plotted in five panels (top to bottom).
(TIF)

S7 Fig NOS in association with gene features. **A**, Nucleosome occupancy scores (NOS) for all datasets (legend on top left) around transcription termination sites (TTS) from our 13,912 gene list used in all analyses. **B**, Codon start sites (CSS). **C**, Codon termination sites (CTS). **D**, Exon start sites (ESS). **E**, Exon termination sites (ETS).
(TIF)

S8 Fig Heatmap of *k*-means clustered groups. Heatmaps of the H1 nucleosome occupancy scores (NOS) around transcription start sites (TSS) grouped by *k*-means clustering. The NOS for each location was divided by the maximum NOS. The median value is 0.201. Expression legend at top and bottom, with arrows denoting TSS.
(TIF)

S9 Fig NOS around *k*-means clustered groups. Nucleosome occupancy scores (NOS) for all 12 datasets around 8 of 10 clusters, see Fig 1D for two others, grouped by *k*-means clustering of the H1 signal around transcription start sites (TSS), see S8 Fig for heatmaps of the groups.
(TIF)

S10 Fig Site of maximum NOS for each cell line per cluster. Location of maximum NOS for all 12 cell lines per cluster around the TSS. Boxes are labelled as per the numbered clusters in Fig 1D and S9 Fig, as defined in S8 Fig, and indicate the region of the maximum NOS for the majority of cell lines for a given cluster.
(TIF)

S11 Fig NOS around chromatin state start sites. On top is a table of chromatin state definitions for the 15 states used in Fig 2F. On bottom, nucleosome occupancy scores (NOS) of the H1 dataset around 15 chromatin state start sites grouped into panels based on similar functional candidate annotations.
(TIF)

S12 Fig FFT of DNA methylation within the core particle. Fast Fourier transforms (FFT) of methylation frequencies within the nucleosome core particle with color coded legend on top.
(TIF)

S13 Fig Transcription factors identified by motif analysis. HOMER identified transcription factor binding motifs within enhancer binding sites, as defined by surrounding nucleosome occupancies (Methods). Their associated gene, along with their q-value is also included.
(TIF)

S1 Table Raw sequencing data metrics. Tables for both H1 and H9 (from top to bottom, respectively) with raw read count and alignment data from each biological replicate, as per Bowtie2.
(DOC)

S2 Table Matrix of H1 bamCorrelate results. Table of Pearson correlation coefficients for next-generation sequencing data for the H1 cell line. This analysis compares all individual H1 replicates to one another, to each sequencing run, and to the pooled H1 dataset. Pooled datasets from the biological replicates are compared to one another and to the pooled H1 dataset.
(DOC)

S3 Table Matrix of H9 bamCorrelate results. Table of Pearson correlation coefficients for next-generation sequencing data for the H9 cell line. This analysis compares all individual H9 replicates to one another, each sequencing run and to the pooled H9 dataset. Pooled datasets from the biological replicates are compared to one another and to the pooled H9 dataset.
(DOC)

S4 Table *P*-values of post-translational modifications. Table of *p*-values generated by Mann-Whitney-Wilcoxon of the effect of histone post-translational modifications on nucleosome occupancy.
(DOC)

S5 Table *P*-values for methylations. Top panel; Mann-Whitney-Wilcoxon *p*-values for all comparisons of the number of 5-methylcytosines found in a nucleosome and its effect on the average nucleosome occupancy. Bottom panel; Mann-Whitney-Wilcoxon *p*-values for all comparisons of the number of 5-hydroxymethylcytosines found in a nucleosome and its effect on the average nucleosome occupancy.
(DOC)
