# Supplementary figures and images for: Nucleosome Organization in Human Embryonic Stem Cells
Source: PLoS One. 2015 Aug 25;10(8):e0136314. doi: 10.1371/journal.pone.0136314 (PMC4549264; doi:10.1371/journal.pone.0136314)

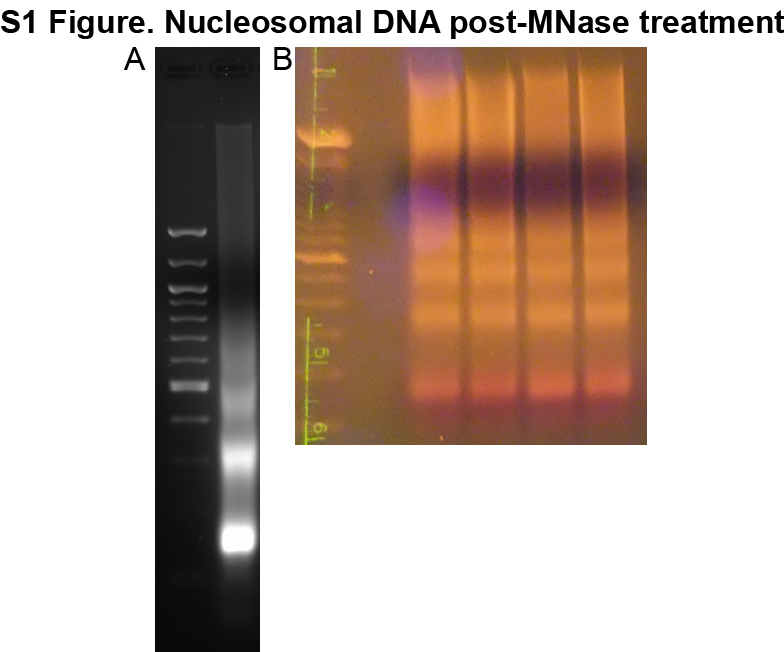

Supplement: S1 Fig — A, MNase treated DNA resolved in a 2% agarose gel stained with ethidium bromide and visualized with a UV light source. B, MNase treated DNA resolved in a 2% agarose gel stained with ethidium bromide and visualized with a visible blue light (DarkReader Transilluminator). (TIF) [file pone.0136314.s001.tif]

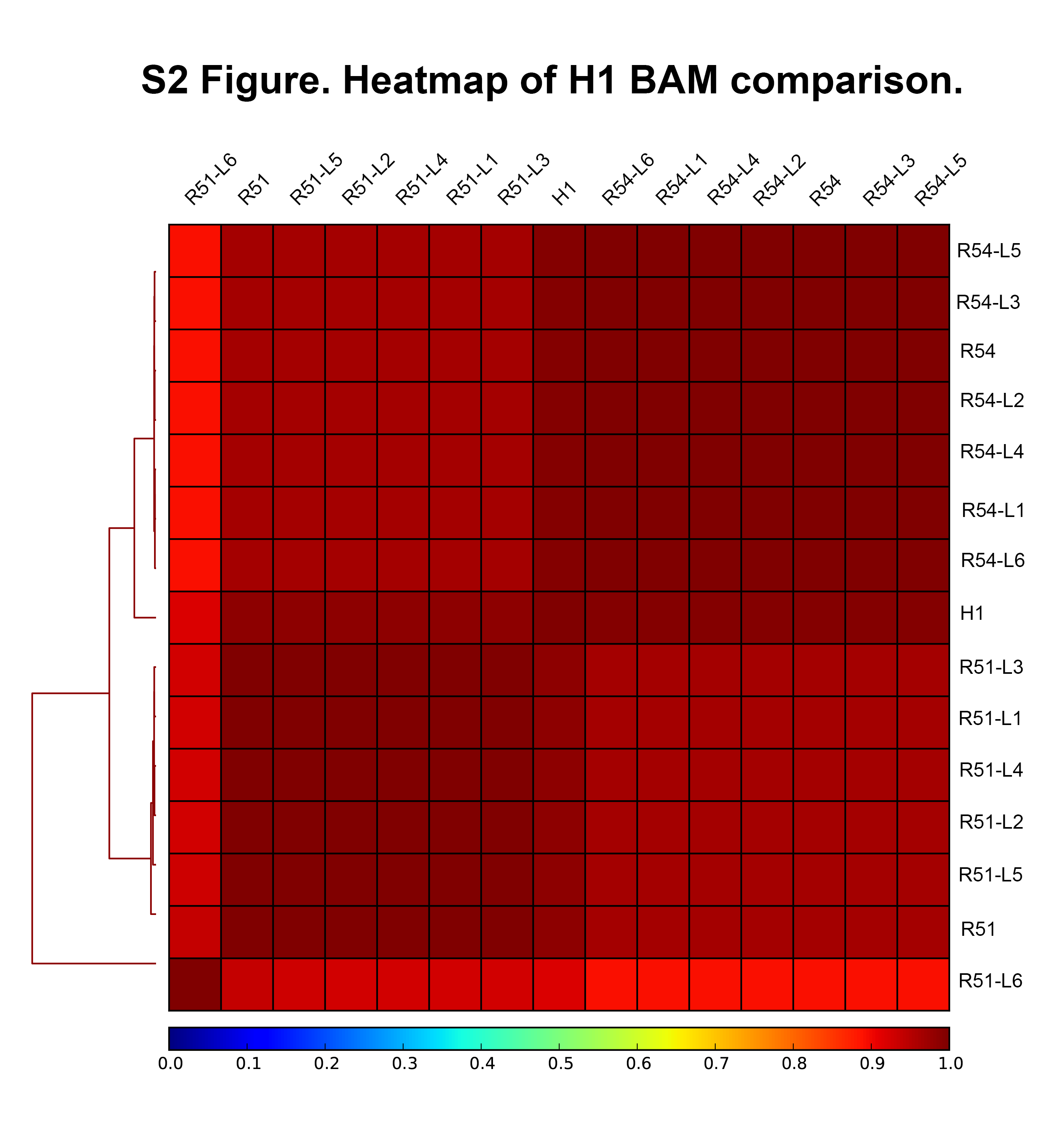

Supplement: S2 Fig — Heatmap visualization with hierarchical clustering of the Pearson correlation coefficients performed for the H1 cell line as per S2 Table. (TIF) [file pone.0136314.s002.tif]

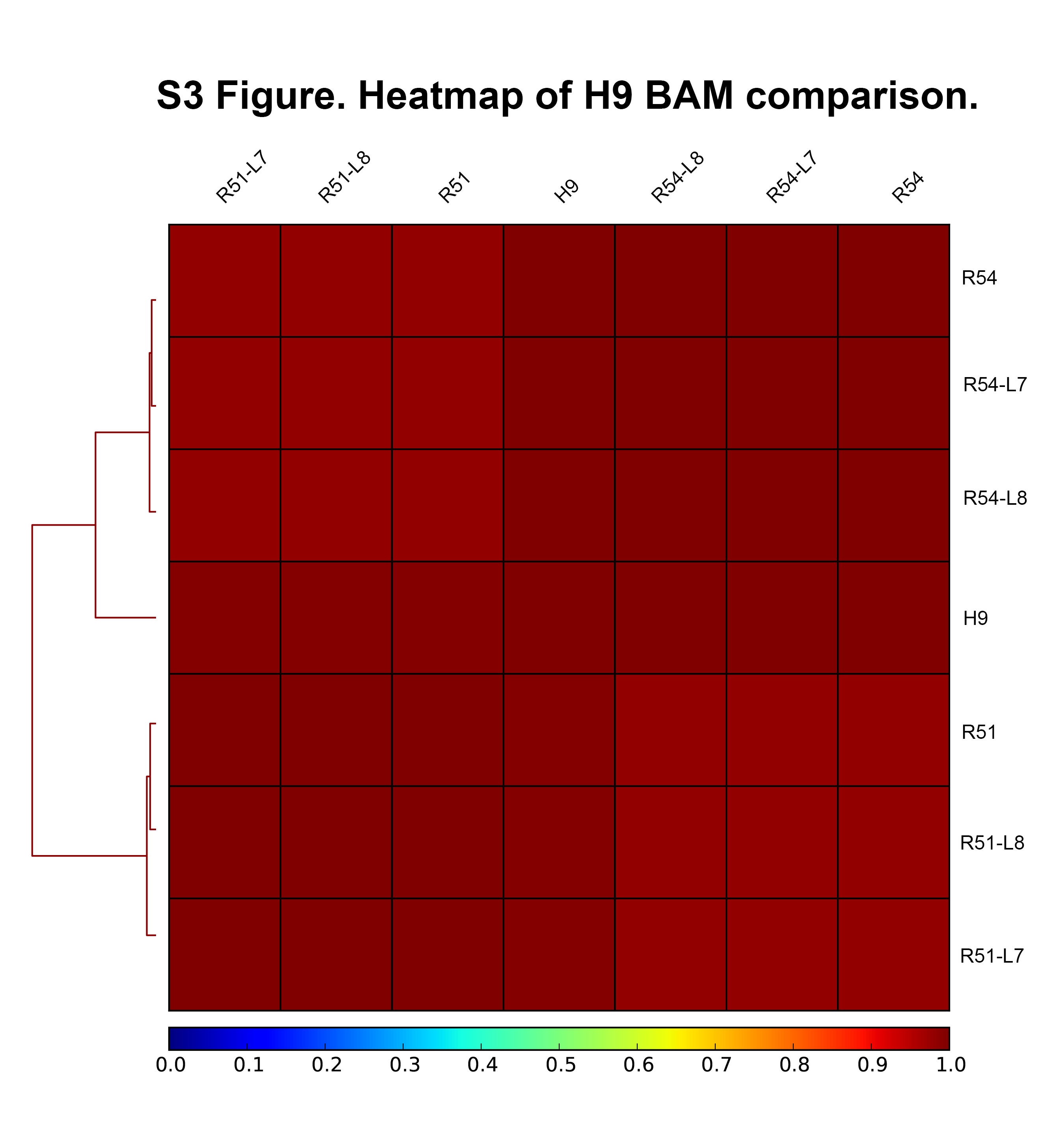

Supplement: S3 Fig — Heatmap visualization with hierarchical clustering of the Pearson correlation coefficients performed for the H9 cell line as per S3 Table. (TIF) [file pone.0136314.s003.tif]

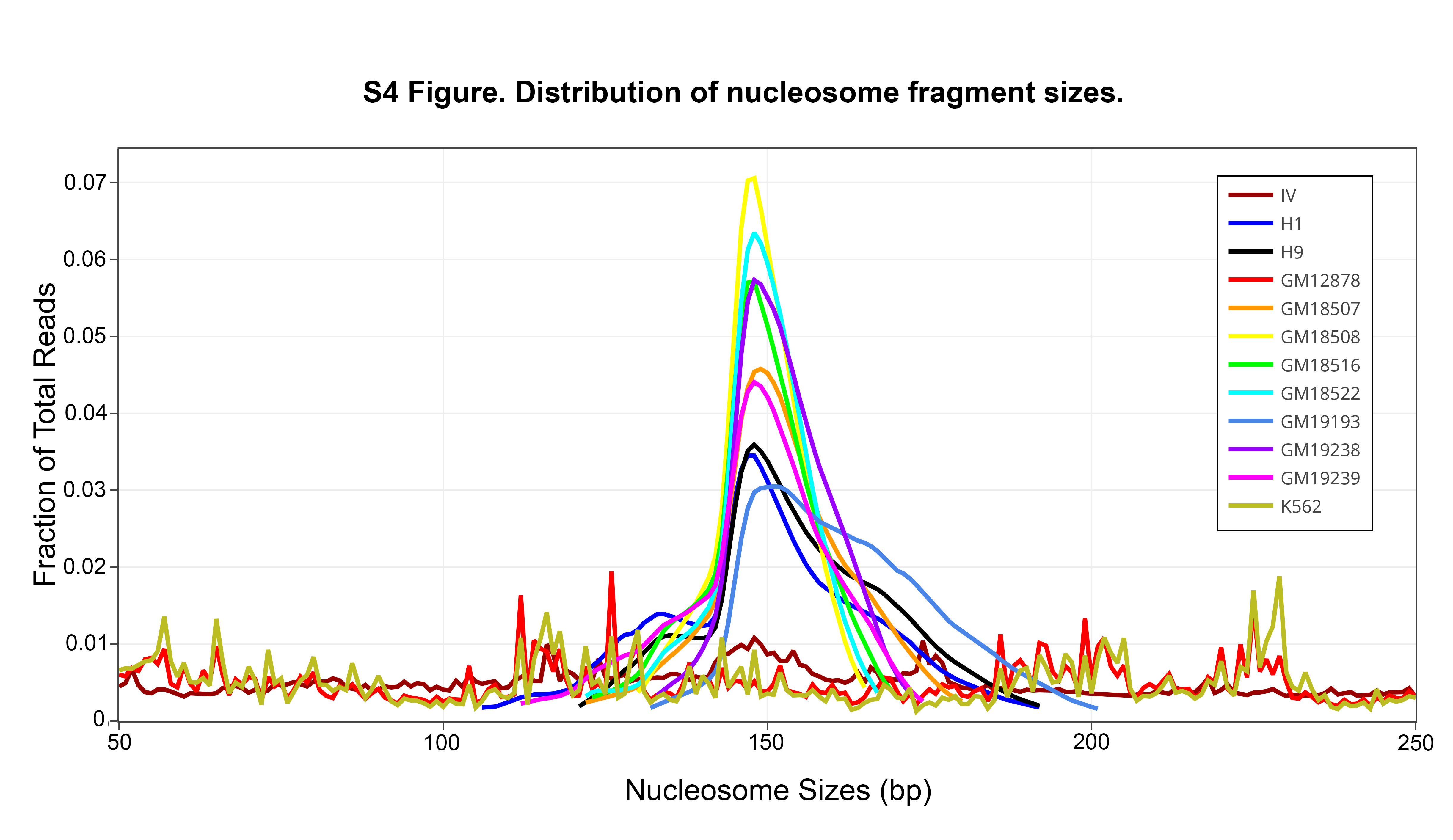

Supplement: S4 Fig — Fragment sizes were inferred through the use of the DANPOS algorithm. (TIF) [file pone.0136314.s004.tif]

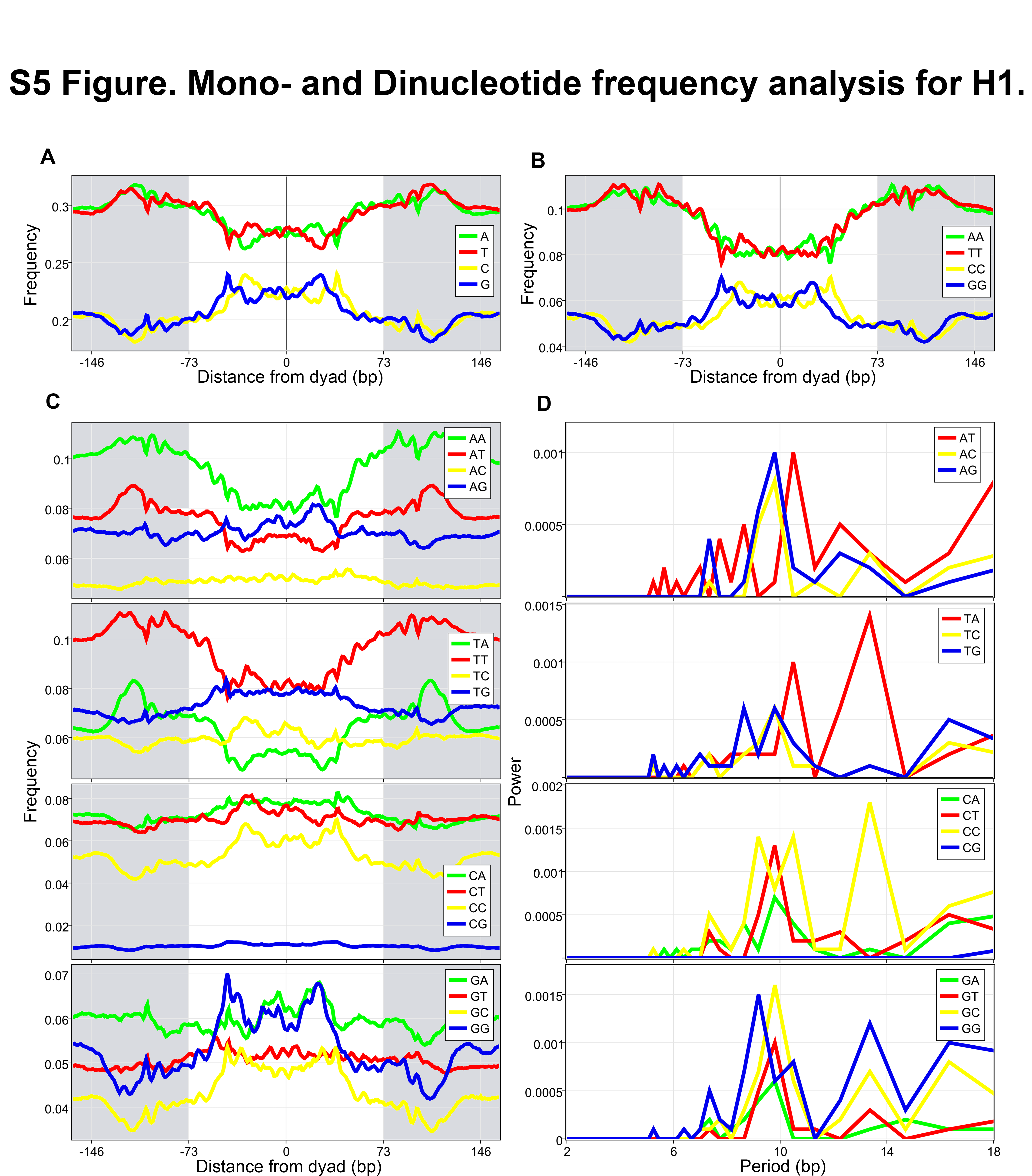

Supplement: S5 Fig — A, Mononucleotide frequencies in relation to the dyad. B, Dinucleotide frequencies for AA, TT, CC, and GG in relation to the dyad. C, Dinucleotide frequencies for all 16 dinucleotides plotted in four panels (top to bottom). D, Fast Fourier transforms (FFT) for all dinucleotides (minus AA and TT, see Fig 1A) plotted in four panels (top to bottom). (TIF) [file pone.0136314.s005.tif]

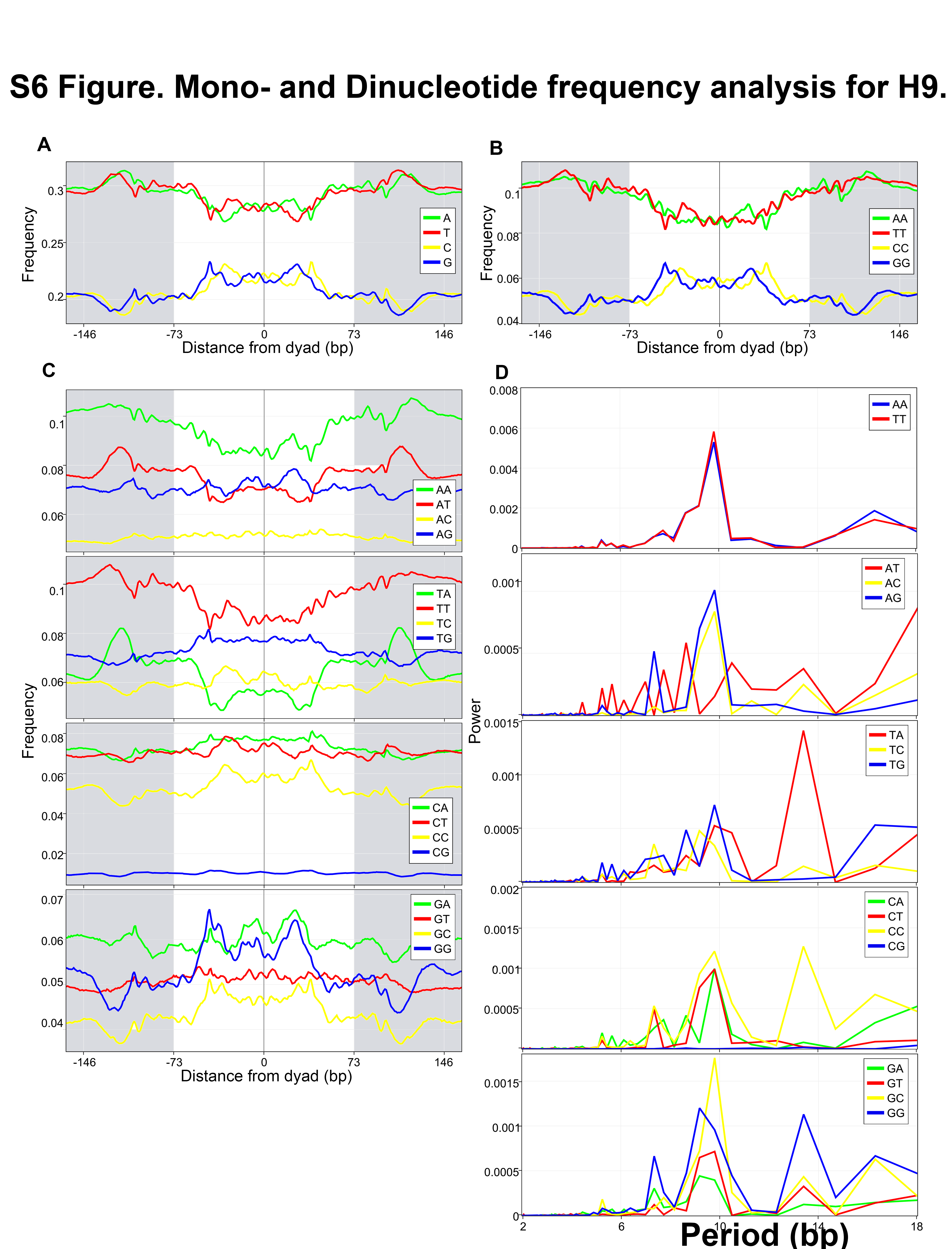

Supplement: S6 Fig — A, Mononucleotide frequencies in relation to the dyad. B, Dinucleotide frequencies for AA, TT, CC, and GG in relation to the dyad. C, Dinucleotide frequencies for all 16 dinucleotides plotted in four panels (top to bottom). D, Fast Fourier transforms (FFT) for all dinucleotides plotted in five panels (top to bottom). (TIF) [file pone.0136314.s006.tif]

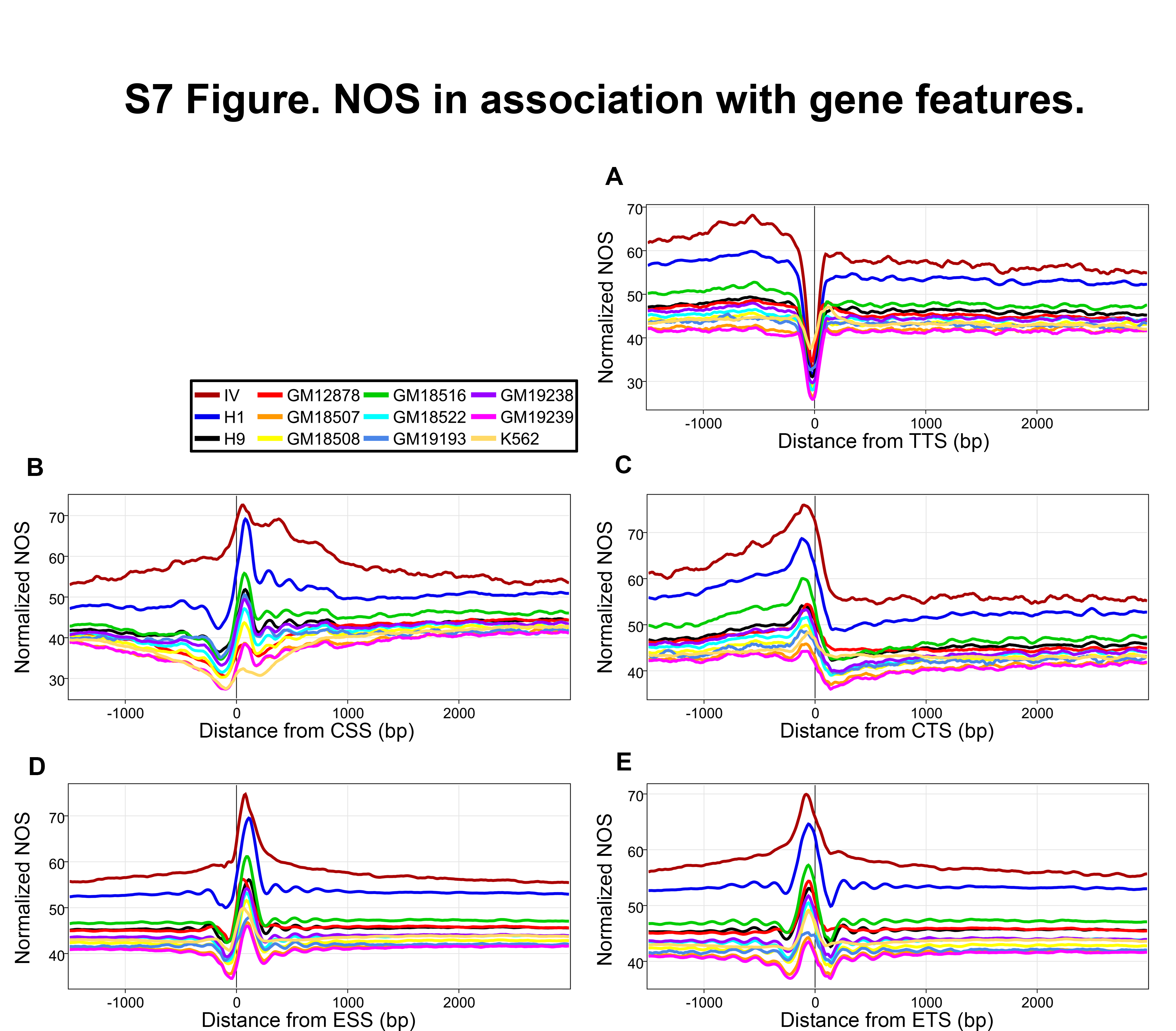

Supplement: S7 Fig — A, Nucleosome occupancy scores (NOS) for all datasets (legend on top left) around transcription termination sites (TTS) from our 13,912 gene list used in all analyses. B, Codon start sites (CSS). C, Codon termination sites (CTS). D, Exon start sites (ESS). E, Exon termination sites (ETS). (TIF) [file pone.0136314.s007.tif]

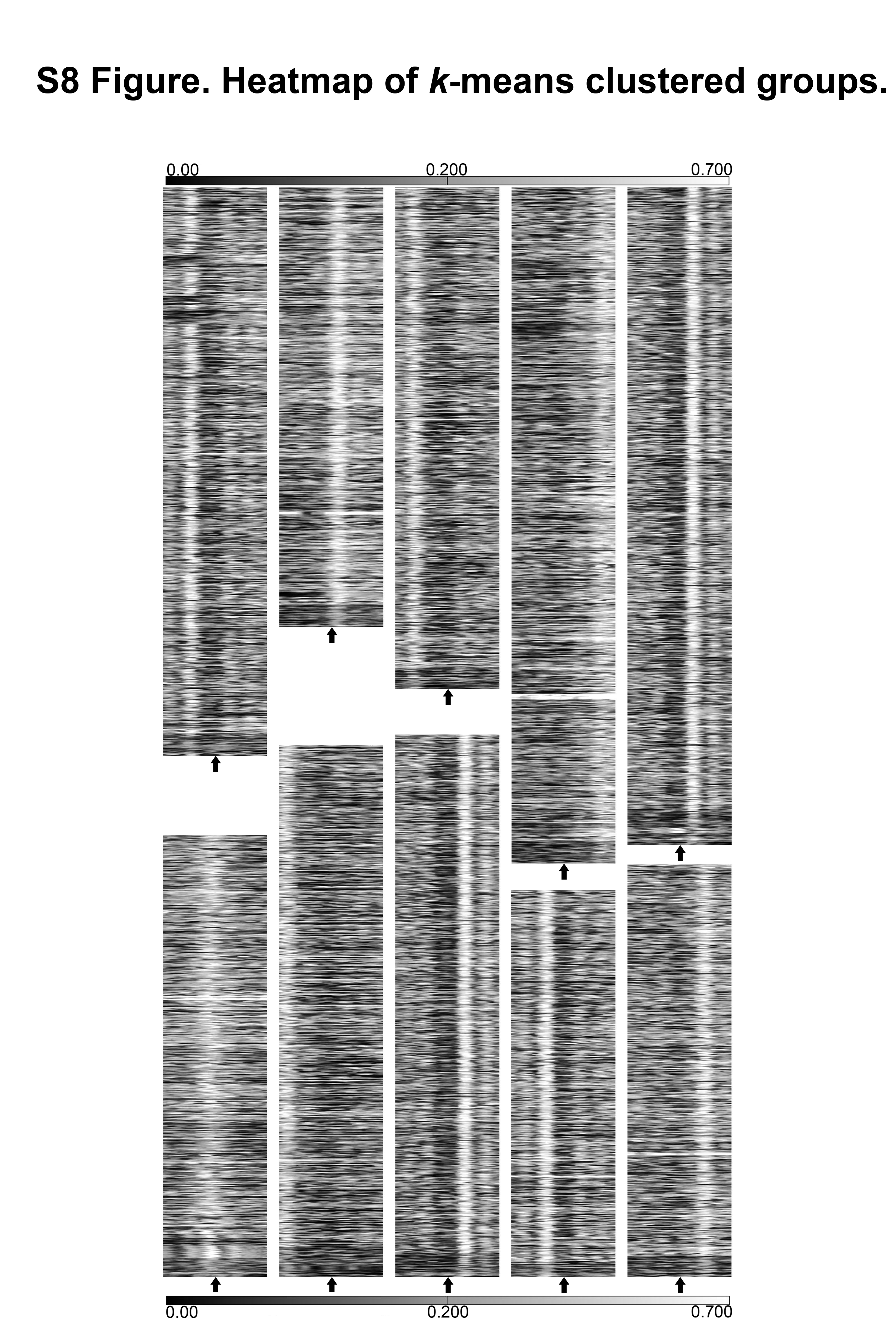

Supplement: S8 Fig — Heatmaps of the H1 nucleosome occupancy scores (NOS) around transcription start sites (TSS) grouped by k-means clustering. The NOS for each location was divided by the maximum NOS. The median value is 0.201. Expression legend at top and bottom, with arrows denoting TSS. (TIF) [file pone.0136314.s008.tif]

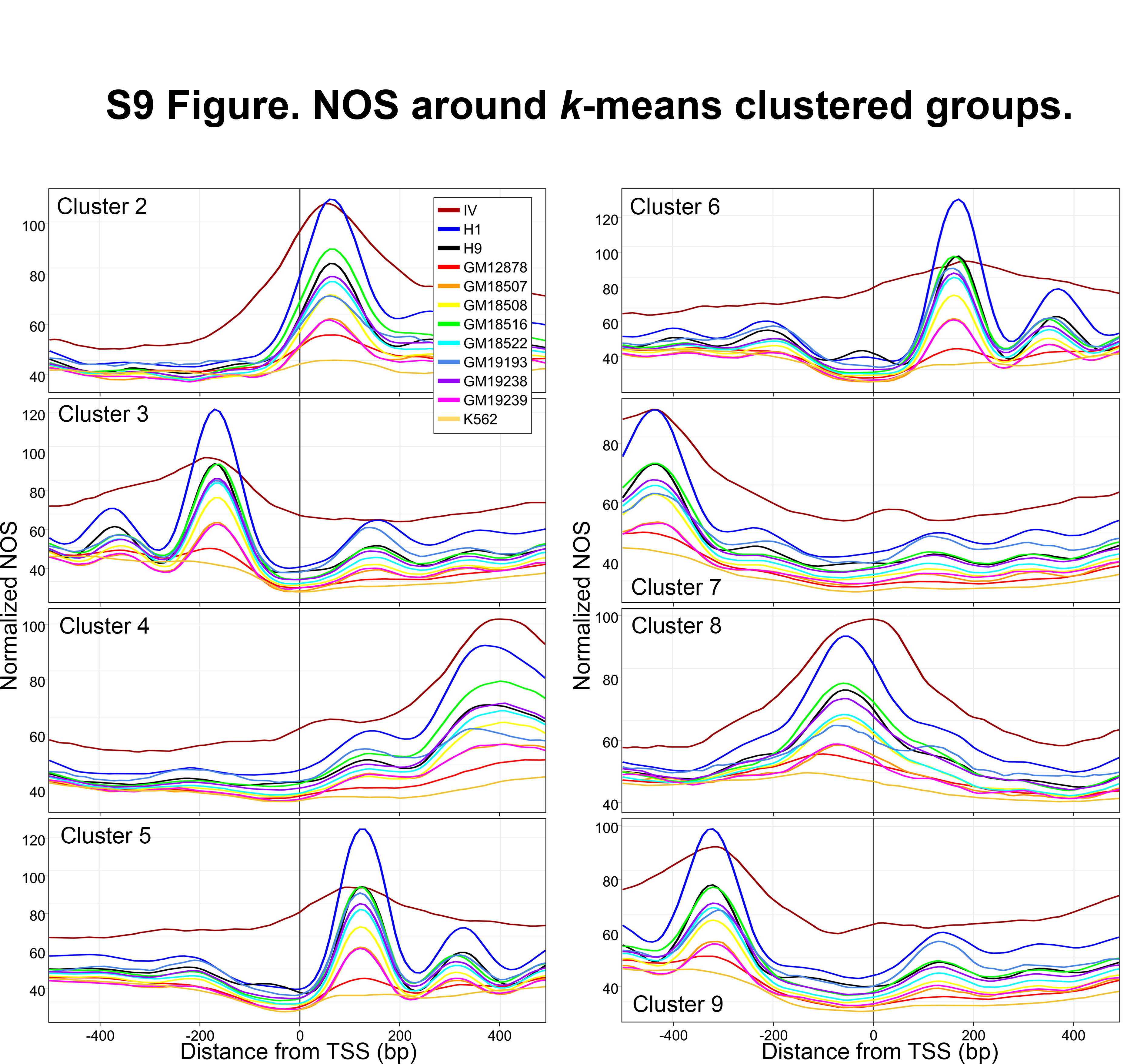

Supplement: S9 Fig — Nucleosome occupancy scores (NOS) for all 12 datasets around 8 of 10 clusters, see Fig 1D for two others, grouped by k-means clustering of the H1 signal around transcription start sites (TSS), see S8 Fig for heatmaps of the groups. (TIF) [file pone.0136314.s009.tif]

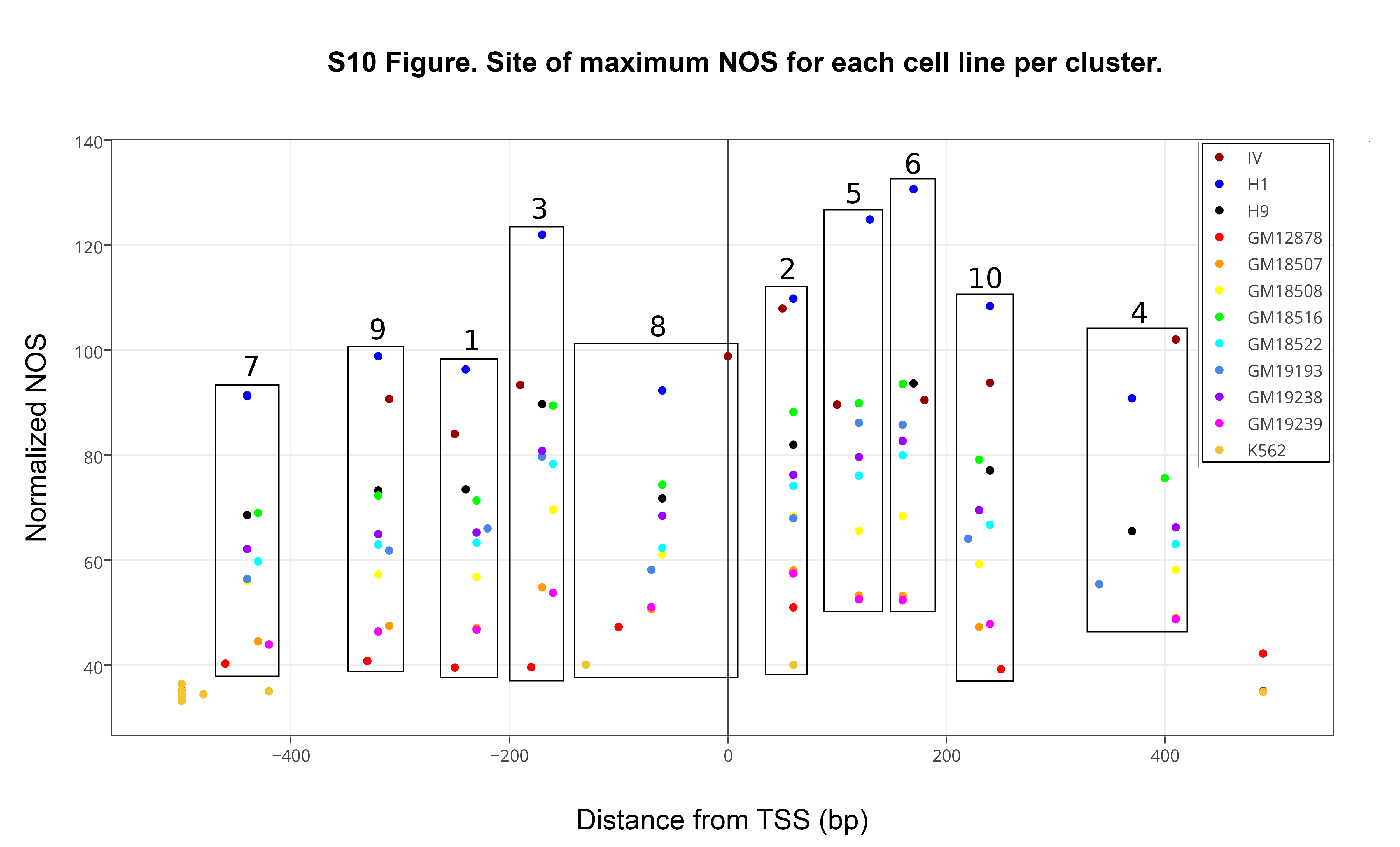

Supplement: S10 Fig — Location of maximum NOS for all 12 cell lines per cluster around the TSS. Boxes are labelled as per the numbered clusters in Fig 1D and S9 Fig, as defined in S8 Fig, and indicate the region of the maximum NOS for the majority of cell lines for a given cluster. (TIF) [file pone.0136314.s010.tif]

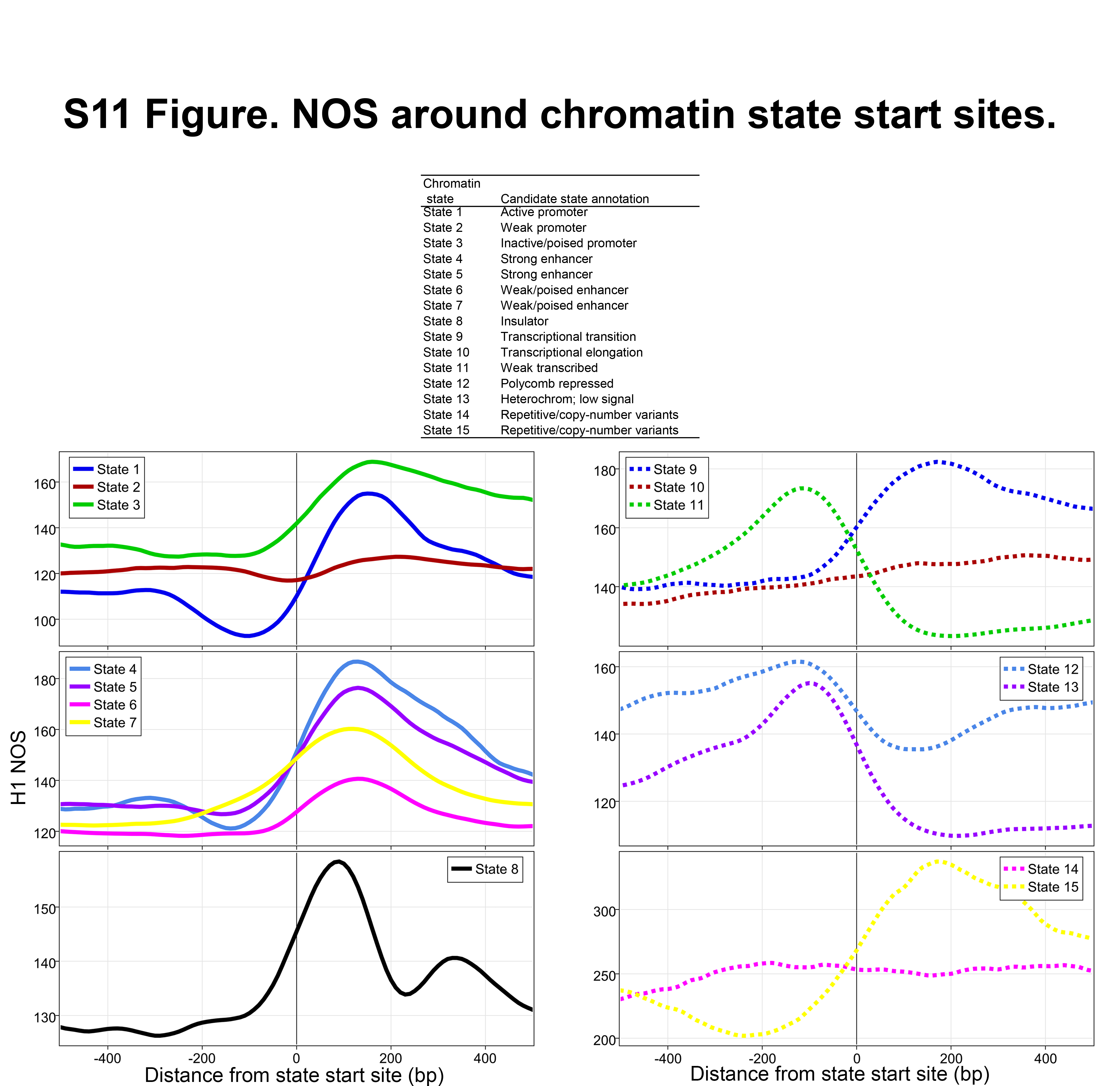

Supplement: S11 Fig — On top is a table of chromatin state definitions for the 15 states used in Fig 2F. On bottom, nucleosome occupancy scores (NOS) of the H1 dataset around 15 chromatin state start sites grouped into panels based on similar functional candidate annotations. (TIF) [file pone.0136314.s011.tif]

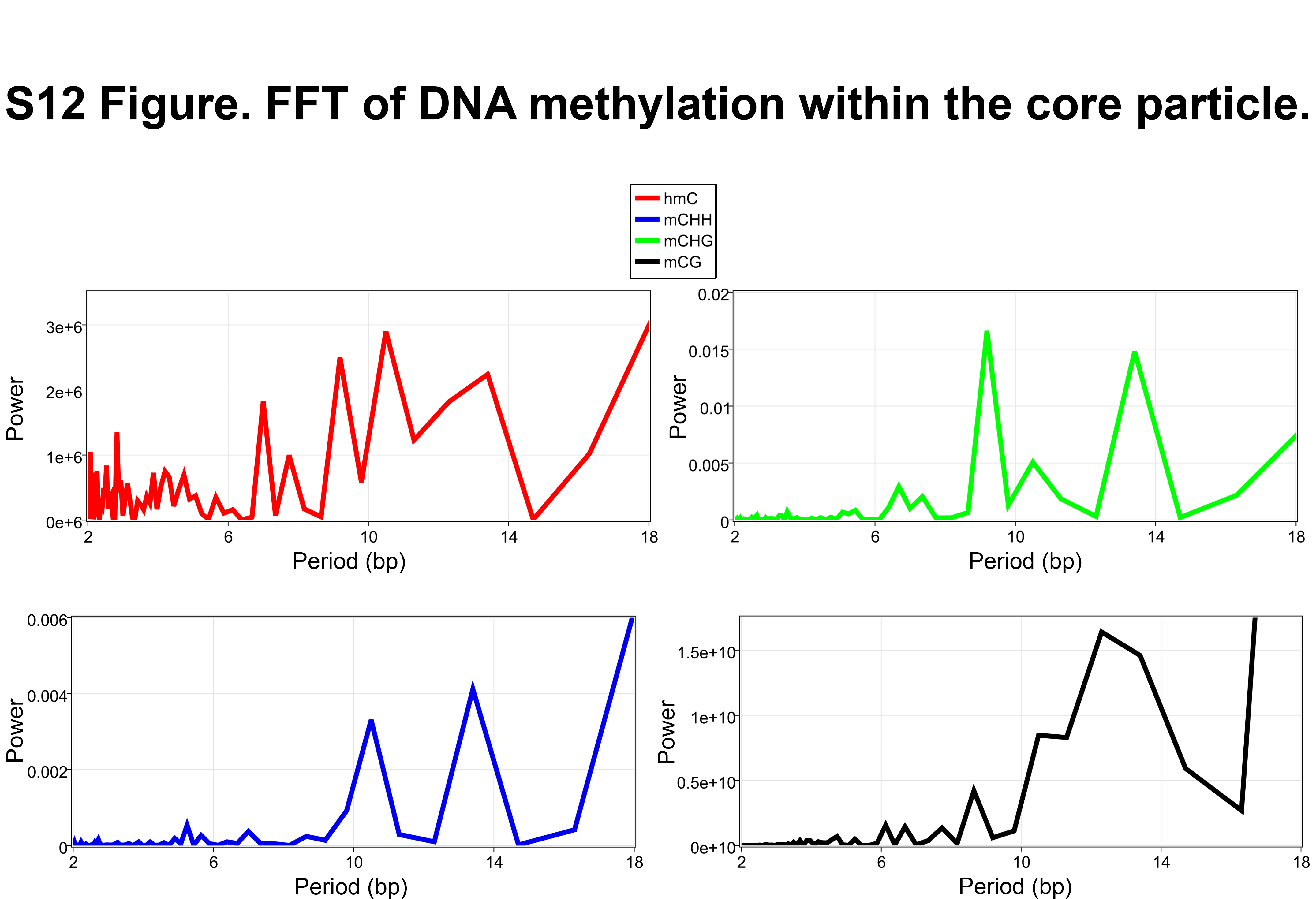

Supplement: S12 Fig — Fast Fourier transforms (FFT) of methylation frequencies within the nucleosome core particle with color coded legend on top. (TIF) [file pone.0136314.s012.tif]

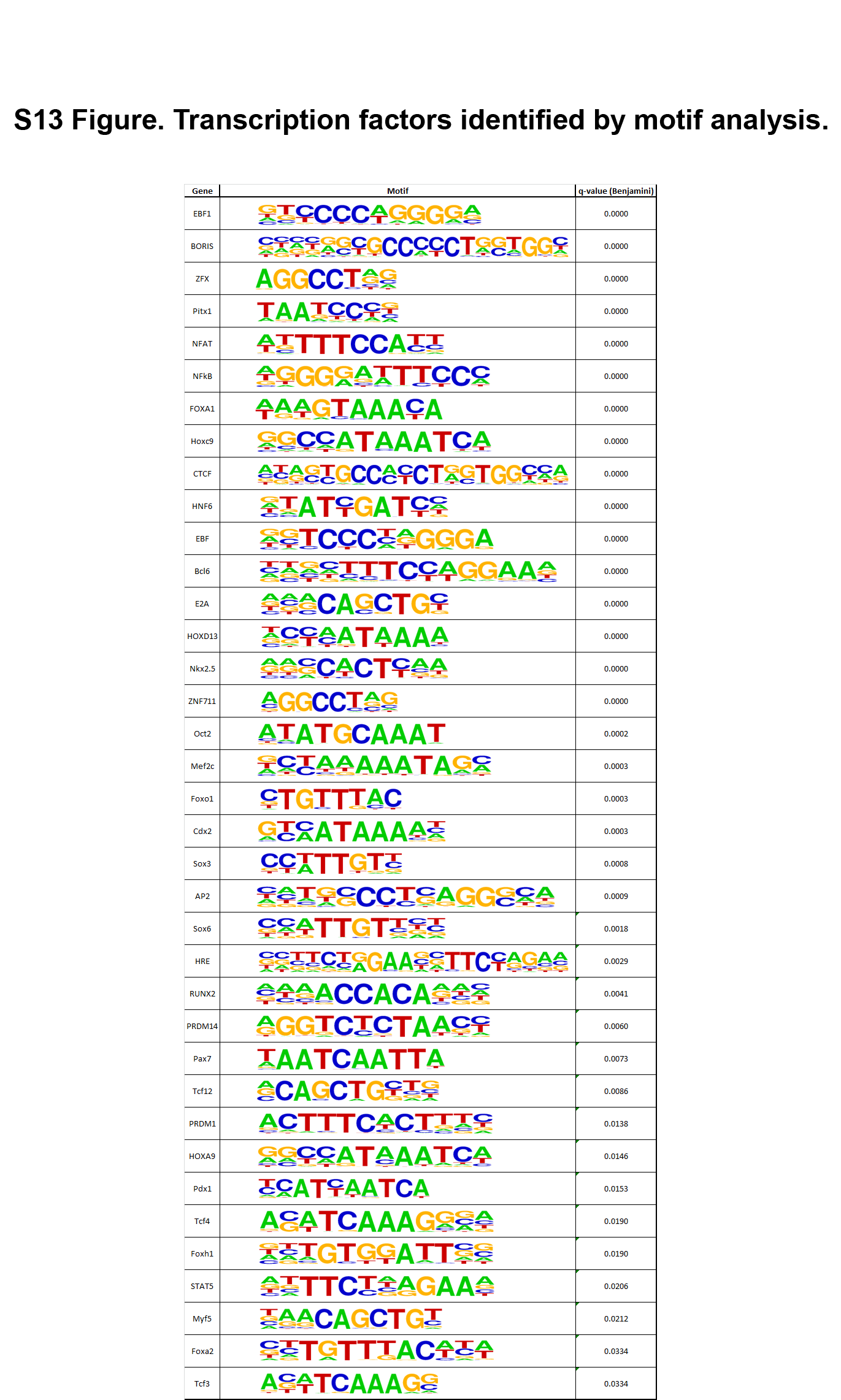

Supplement: S13 Fig — HOMER identified transcription factor binding motifs within enhancer binding sites, as defined by surrounding nucleosome occupancies (Methods). Their associated gene, along with their q-value is also included. (TIF) [file pone.0136314.s013.tif]
