# Supplementary material for: Nucleosome Organization in Human Embryonic Stem Cells
Source: PLoS One. 2015 Aug 25;10(8):e0136314. doi: 10.1371/journal.pone.0136314 (PMC4549264; doi:10.1371/journal.pone.0136314)
Supplement: S1 Table — Tables for both H1 and H9 (from top to bottom, respectively) with raw read count and alignment data from each biological replicate, as per Bowtie2. (DOC) [file pone.0136314.s014.doc]

**S1 Table. Raw Sequencing data metrics.**

| **Run-Sample** | **Raw Read Count** | **Aligned Reads** | **Percent Alignment** |
| --- | --- | --- | --- |
| R51-H1 | 1170766893 | 969768800 | 82.83% |
| R54-H1 | 1416058758 | 1109924496 | 78.38% |
| H1 | 2586825651 | 2079693296 | 80.40% |
|  |  |  |  |
| **Run-Sample** | **Raw Read Count** | **Aligned Reads** | **Percent Alignment** |
| R51-H9 | 408853114 | 376082908 | 91.98% |
| R54-H9 | 460465813 | 371384880 | 80.65% |
| H9 | 869318927 | 747467788 | 85.98% |
