# Supplementary material for: Nucleosome Organization in Human Embryonic Stem Cells
Source: PLoS One. 2015 Aug 25;10(8):e0136314. doi: 10.1371/journal.pone.0136314 (PMC4549264; doi:10.1371/journal.pone.0136314)
Supplement: S2 Table — Table of Pearson correlation coefficients for next-generation sequencing data for the H1 cell line. This analysis compares all individual H1 replicates to one another, to each sequencing run, and to the pooled H1 dataset. Pooled datasets from the biological replicates are compared to one another and to the pooled H1 dataset. (DOC) [file pone.0136314.s015.doc]

**S2 Table. Matrix of H1 bamCorrelate results.**

|  | **R51-L1** | **R51-L2** | **R51-L3** | **R51-L4** | **R51-L5** | **R51-L6** | **R54-L1** | **R54-L2** | **R54-L3** | **R54-L4** | **R54-L5** | **R54-L6** | **R51** | **R54** | **H1** |
| --- | --- | --- | --- | --- | --- | --- | --- | --- | --- | --- | --- | --- | --- | --- | --- |
| **R51-L1** | 1 | 0.9967 | 0.9967 | 0.9967 | 0.9962 | 0.9287 | 0.9673 | 0.9678 | 0.9679 | 0.9669 | 0.9675 | 0.9671 | 0.9982 | 0.9683 | 0.9869 |
| **R51-L2** | 0.9967 | 1 | 0.9967 | 0.9971 | 0.9962 | 0.9274 | 0.9672 | 0.9676 | 0.9675 | 0.9669 | 0.9671 | 0.967 | 0.9983 | 0.9682 | 0.9869 |
| **R51-L3** | 0.9967 | 0.9967 | 1 | 0.9967 | 0.9964 | 0.9287 | 0.9673 | 0.9679 | 0.9679 | 0.967 | 0.9676 | 0.9672 | 0.9983 | 0.9684 | 0.987 |
| **R51-L4** | 0.9967 | 0.9971 | 0.9967 | 1 | 0.9962 | 0.927 | 0.9675 | 0.9678 | 0.9677 | 0.9672 | 0.9674 | 0.9672 | 0.9983 | 0.9684 | 0.987 |
| **R51-L5** | 0.9962 | 0.9962 | 0.9964 | 0.9962 | 1 | 0.9298 | 0.9654 | 0.966 | 0.9661 | 0.965 | 0.9657 | 0.9652 | 0.9981 | 0.9665 | 0.9857 |
| **R51-L6** | 0.9287 | 0.9274 | 0.9287 | 0.927 | 0.9298 | 1 | 0.8927 | 0.8932 | 0.8941 | 0.8925 | 0.8935 | 0.8923 | 0.9397 | 0.8939 | 0.918 |
| **R54-L1** | 0.9673 | 0.9672 | 0.9673 | 0.9675 | 0.9654 | 0.8927 | 1 | 0.9979 | 0.9974 | 0.998 | 0.9975 | 0.9979 | 0.9673 | 0.9991 | 0.9946 |
| **R54-L2** | 0.9678 | 0.9676 | 0.9679 | 0.9678 | 0.966 | 0.8932 | 0.9979 | 1 | 0.9974 | 0.9978 | 0.9975 | 0.9978 | 0.9678 | 0.9991 | 0.9947 |
| **R54-L3** | 0.9679 | 0.9675 | 0.9679 | 0.9677 | 0.9661 | 0.8941 | 0.9974 | 0.9974 | 1 | 0.9972 | 0.9973 | 0.9974 | 0.9679 | 0.9987 | 0.9945 |
| **R54-L4** | 0.9669 | 0.9669 | 0.967 | 0.9672 | 0.965 | 0.8925 | 0.998 | 0.9978 | 0.9972 | 1 | 0.9974 | 0.9979 | 0.967 | 0.9991 | 0.9944 |
| **R54-L5** | 0.9675 | 0.9671 | 0.9676 | 0.9674 | 0.9657 | 0.8935 | 0.9975 | 0.9975 | 0.9973 | 0.9974 | 1 | 0.9975 | 0.9675 | 0.9988 | 0.9944 |
| **R54-L6** | 0.9671 | 0.967 | 0.9672 | 0.9672 | 0.9652 | 0.8923 | 0.9979 | 0.9978 | 0.9974 | 0.9979 | 0.9975 | 1 | 0.9671 | 0.9991 | 0.9945 |
| **R51** | 0.9982 | 0.9983 | 0.9983 | 0.9983 | 0.9981 | 0.9397 | 0.9673 | 0.9678 | 0.9679 | 0.967 | 0.9675 | 0.9671 | 1 | 0.9684 | 0.9876 |
| **R54** | 0.9683 | 0.9682 | 0.9684 | 0.9684 | 0.9665 | 0.8939 | 0.9991 | 0.9991 | 0.9987 | 0.9991 | 0.9988 | 0.9991 | 0.9684 | 1 | 0.9955 |
| **H1** | 0.9869 | 0.9869 | 0.987 | 0.987 | 0.9857 | 0.918 | 0.9946 | 0.9947 | 0.9945 | 0.9944 | 0.9944 | 0.9945 | 0.9876 | 0.9955 | 1 |
