# Supplementary material for: Nucleosome Organization in Human Embryonic Stem Cells
Source: PLoS One. 2015 Aug 25;10(8):e0136314. doi: 10.1371/journal.pone.0136314 (PMC4549264; doi:10.1371/journal.pone.0136314)
Supplement: S4 Table — Table of p-values generated by Mann-Whitney-Wilcoxon of the effect of histone post-translational modifications on nucleosome occupancy. (DOC) [file pone.0136314.s017.doc]

**S4 Table. *P*-values of post-translational modifications.**

|  | H3K4me1 | H3K4me2 | H3K4me3 | H3K9ac | H3K9me3 | H4K20me1 | H3K27ac | H3K27me3 | H3K36me3 | H3K79me2 |
| --- | --- | --- | --- | --- | --- | --- | --- | --- | --- | --- |
| H3K4me1 | - | 2.20E-16* | 4.80E-02 | 2.20E-16 | 2.20E-16 | 2.81E-02 | 2.20E-16 | 2.20E-16 | 7.71E-14 | 2.20E-16 |
| H3K4me2 | - | - | 2.20E-16 | 2.20E-16 | 2.20E-16 | 2.20E-16 | 2.20E-16 | 2.20E-16 | 5.70E-16 | 2.20E-16 |
| H3K4me3 | - | - | - | 2.20E-16 | 2.20E-16 | 1.77E-02 | 8.70E-10 | 2.20E-16 | 1.53E-12 | 2.20E-16 |
| H3K9ac | - | - | - | - | 2.20E-16 | 2.20E-16 | 2.20E-16 | 2.20E-16 | 2.20E-16 | 2.20E-16 |
| H3K9me3 | - | - | - | - | - | 2.20E-16 | 2.20E-16 | 2.20E-16 | 2.20E-16 | 9.13E-02 |
| H4K20me1 | - | - | - | - | - | - | 2.20E-16 | 2.20E-16 | 1.05E-06 | 2.20E-16 |
| H3K27ac | - | - | - | - | - | - | - | 1.54E-11 | 2.20E-16 | 2.20E-16 |
| H3K27me3 | - | - | - | - | - | - | - | - | 2.20E-16 | 2.20E-16 |
| H3K36me3 | - | - | - | - | - | - | - | - | - | 2.20E-16 |
| H3K79me2 | - | - | - | - | - | - | - | - | - | - |
| *Mann-Whitney-Wilcoxon *p*-values | | | |  |  |  |  |  |  |  |
