# Supplementary material for: Nucleosome Organization in Human Embryonic Stem Cells
Source: PLoS One. 2015 Aug 25;10(8):e0136314. doi: 10.1371/journal.pone.0136314 (PMC4549264; doi:10.1371/journal.pone.0136314)
Supplement: S5 Table — Top panel; Mann-Whitney-Wilcoxon p-values for all comparisons of the number of 5-methylcytosines found in a nucleosome and its effect on the average nucleosome occupancy. Bottom panel; Mann-Whitney-Wilcoxon p-values for all comparisons of the number of 5-hydroxymethylcytosines found in a nucleosome and its effect on the average nucleosome occupancy. (DOC) [file pone.0136314.s018.doc]

**S5 Table. *P*-values for methylations.**

|  | Zero | One | Two | Three | Four | Five | Six + |
| --- | --- | --- | --- | --- | --- | --- | --- |
| Zero | - | 2.20E-16* | 2.20E-16 | 2.20E-16 | 2.20E-16 | 2.20E-16 | 2.20E-16 |
| One | - | - | 2.20E-16 | 2.20E-16 | 2.20E-16 | 2.20E-16 | 2.20E-16 |
| Two | - | - | - | 2.20E-16 | 2.20E-16 | 2.20E-16 | 2.20E-16 |
| Three | - | - | - | - | 2.20E-16 | 2.20E-16 | 2.20E-16 |
| Four | - | - | - | - | - | 2.20E-16 | 2.20E-16 |
| Five | - | - | - | - | - | - | 2.20E-16 |
| Six + | - | - | - | - | - | - | - |
| *Mann-Whitney-Wilcoxon *p*-values | | | |  |  |  |  |
|  |  |  |  |  |  |  |  |
|  | Zero | One | Two | Three | Four + |  |  |
| Zero | - | 2.20E-16* | 2.20E-16 | 2.20E-16 | 2.20E-16 |  |  |
| One | - | - | 2.20E-16 | 2.20E-16 | 2.20E-16 |  |  |
| Two | - | - | - | 2.20E-16 | 2.20E-16 |  |  |
| Three | - | - | - | - | 1.40E-07 |  |  |
| Four + | - | - | - | - | - |  |  |
| *Mann-Whitney-Wilcoxon *p*-values | | | |  |  |  |  |
